# Selective Regimes and Evolutionary Dynamics of Z and W Gametologs Across an Expanded Avian Neo-Sex Chromosome

**DOI:** 10.1093/gbe/evag204

**Published:** 2026-08-10

**Authors:** Simon J Ellerstrand, Bengt Hansson

**Affiliations:** Department of Biology, Lund University, Ecology Building, 223 62 Lund, Sweden; Department of Biology, Lund University, Ecology Building, 223 62 Lund, Sweden; Science for Life Laboratory (SciLifeLab), Lund University, Ecology Building, 223 62 Lund, Sweden; BECC—Biodiversity and Ecosystem services in a Changing Climate, Lund University, Ecology Building, 223 62 Lund, Sweden

**Keywords:** sex chromosome evolution, recombination cessation, evolutionary strata, hemizygous selection, gene essentiality, *Alauda*

## Abstract

Sex chromosome evolution is characterized by Y/W degeneration and its consequences for selection on X/Z-linked recessive mutations in the heterogametic sex, yet how functional constraints modulate the evolutionary dynamics of Z–W gametologs remains poorly resolved. We addressed this question in larks (Alaudidae), a lineage carrying enlarged neo-sex chromosomes with multiple evolutionary strata formed through repeated translocations and successive recombination-suppression events. Using whole-genome sequences of males and females of two *Alauda* species, we analysed 1,759 Z–W gametologs. We found that W-gene degeneration was governed primarily by evolutionary time and intrinsic gene features: long genes were lost earliest, although this tendency was mitigated by gene essentiality, as indicated by high haploinsufficiency scores. Z-linked genes lacking a functional W counterpart exhibited higher nonsynonymous divergence than Z genes retaining a functional W, consistent with selection-driven faster-Z evolution. The strongest signature of purifying selection, comparable to that of pseudoautosomal genes, was observed in haploinsufficient Z-linked genes, which were also more likely to retain W gametologs. Although W genes generally diverged faster, their selection signatures covaried with those of Z and with haploinsufficiency, suggesting partially shared Z–W evolutionary dynamics. Notably, a small subset of W genes showed intensified or positive selection, including several genes associated with putative female-specific functions. Together, our results demonstrate that gene essentiality slows functional divergence on both Z and W, modulates faster-Z dynamics and enables key W genes to remain functional—and potentially advantageous for females—for millions of generations after recombination cessation.

Significance Neo-sex chromosomes offer a powerful opportunity to dissect how degeneration, selection and gene function interact over evolutionary time. By studying larks with massively expanded neo-sex chromosomes spanning multiple evolutionary strata, we reveal that gene essentiality plays a central role in shaping both Z- and W-chromosome evolution. Essential genes were more likely to retain functional W copies, and experienced strong purifying selection on the Z, resembling the selective regime of pseudoautosomal genes. In contrast, Z-linked genes that had lost their W counterpart showed elevated functional divergence. These patterns are consistent with selection-driven faster-Z evolution. Although most W genes experienced relaxed selection, a subset showed evidence of intensified or positive selection, including genes with putative female-specific functions. Our results demonstrate that gene essentiality modulates W-gene degeneration, Z–W functional divergence and faster-Z dynamics, providing new insight into the evolutionary dynamics of sex chromosomes over millions of years.

## Introduction

Sex chromosomes repeatedly evolve from autosomes following the acquisition of sex-determining mutations (Ohno 1967). Recombination suppression often develops around these loci and is generally thought to arise through selection favoring tighter linkage between sex-determining and sexually antagonistic alleles (Fisher 1931; Bull 1983; Rice 1987). Suppression may occur abruptly through structural changes such as inversions or spread gradually through local genomic changes, generating evolutionary strata with distinct levels of divergence between the sex-linked haplotypes (Lahn and Page 1999; Xu et al. 2019). Such strata are a recurrent feature of sex chromosome evolution across diverse taxa, including mammals, birds, fish, plants, fungi, mosses and algae (Bergero et al. 2007; Votintseva and Filatov 2009; McDaniel et al. 2013; Bellott et al. 2014; Bellott et al. 2014; Cortez et al. 2014; Cortez et al. 2014; Zhou et al. 2014; Xu et al. 2019; Peichel et al. 2020).

In many taxa, recombination has ceased across most of the sex chromosomes, leaving only a small pseudoautosomal region (PAR) required for proper meiotic segregation (Graves et al. 1998; Otto et al. 2011; Zhou et al. 2014). Recombination cessation, skewed transmission and reduced effective population size increase the effects of genetic drift, background selection and selective sweeps on sex-linked regions (Hill and Robertson 1966; Smith and Haigh 1974; Charlesworth et al. 1993; Ellegren and Galtier 2016). These effects are strongest on Y/W chromosomes, which lack recombination entirely and therefore tend to accumulate deleterious mutations through Muller's ratchet (Muller 1964). As recombination ceases, homologous genes on the X/Z and Y/W chromosomes (gametologs) diverge, and in most systems the Y/W gametologs gradually degenerate relative to their X/Z counterparts because selection becomes less efficient and genetic drift more pronounced (Bachtrog 2008). Only very rarely do Y/W-linked genes persist after loss of their X/Z gametologs, as in the mammalian gene *PRSSLY* (Hughes et al. 2022).

A common pattern in sex chromosome evolution is faster-X/Z evolution, where X/Z-linked genes evolve more rapidly than autosomal genes (Charlesworth et al. 1987; Mank et al. 2010b). This has been reported in several systems, including mammals, birds and Lepidoptera (Torgerson and Singh 2003; Baines and Harr 2007; Mank et al. 2010a; Sackton et al. 2014), but other studies have found similar or even slower rates on sex chromosomes (Counterman et al. 2004; Vicoso et al. 2008; Rousselle et al. 2016; Mongue et al. 2022; Baird et al. 2025). These contrasting patterns likely reflect differences between lineages in selective regime, effective population size, degree of Y/W degeneration, dosage compensation, general recombination rate (Naurin et al. 2010; Ellegren 2011; Bachtrog 2013), and the distribution of beneficial versus deleterious mutations affecting sex-linked loci (Charlesworth et al. 1987; Vicoso and Charlesworth 2009).

A key consequence of Y/W degeneration is increased exposure of recessive mutations on the X/Z chromosome in the heterogametic sex, which effectively becomes hemizygous. This can increase the efficacy of positive selection on recessive beneficial mutations, as well as strengthen purifying selection against recessive deleterious alleles. Accordingly, genes lacking a functional Y/W gametolog may experience either faster or slower evolution than autosomal genes, depending on the balance between positive and purifying selection (Charlesworth et al. 1987; Vicoso and Charlesworth 2009; Mank et al. 2010b). This effect is typically expected in older sex chromosome systems, where the majority of X/Z genes are hemizygous. In contrast, in younger systems with largely nondegenerated Y/W chromosomes, beneficial recessive mutations on X/Z gametologs may remain masked by retained Y/W gametologs, thereby limiting the efficacy of selection (Mrnjavac et al. 2023). Consistent with this, genes on the *Drosophila miranda* neo-X chromosome show faster nonsynonymous divergence when the Y copy is degenerated than when it remains functional (Zhou and Bachtrog 2012b). Another key consequence of sex chromosome evolution is the smaller effective population size of the X/Z chromosome relative to autosomes—irrespectively of whether the Y/W gametolog remains functional or not—which simultaneously reduces the selection efficiency on X/Z-linked genes, potentially allowing slightly deleterious alleles to raise in frequency through genetic drift (Vicoso and Charlesworth 2009; Mank et al. 2010b). In mammals and *Drosophila* several studies suggest that positive selection-driven faster-X is dominating (Torgerson and Singh 2003; Baines and Harr 2007; Mank et al. 2010b; Zhou and Bachtrog 2012b), while in birds drift-driven faster-Z is often invoked, potentially amplified by male-biased sexual selection that further reduces the effective population size of the Z chromosome (Mank et al. 2010a; Wright et al. 2015; Xu et al. 2019; Hayes et al. 2020; Chase et al. 2025).

The outcome of sex-linkage may also be modulated by gene-specific properties including essentiality, length and sex-specific function (Lipman et al. 2002; Bachtrog 2013; Bellott et al. 2014, 2017; Soni and Eyre-Walker 2022). How various gene properties shape faster-X/Z evolution remains less well understood. For example, longer genes constitute a larger mutational target and are thus believed more difficult to maintain than shorter genes, thereby affecting the predicted outcome of both Y/W degeneration and efficacy of selection on X/Z gametologs. Furthermore, genes that are essential, broadly expressed and dosage sensitive are more likely to be retained on both sex chromosomes and to remain evolutionarily conserved regardless of genomic location (Bellott et al. 2014, 2017; Wright et al. 2014; Peichel et al. 2020). Thus, such genes should follow similar evolutionary trajectories when X/Z-linked as when autosomal and should be less likely to lose their Y/W gametologs. By contrast, less essential genes may be more likely to lose their Y/W gametologs, experience weaker purifying selection and greater evolutionary flexibility. Such genes may accumulate slightly deleterious mutations more readily, evolve at a faster pace and occasionally undergo adaptive changes—a combination of processes that can contribute to selection-driven faster-X/Z evolution (Ellegren 2009; Mank et al. 2010b; Meisel 2022). Consistent with this, Xu et al. (2019) found that avian Z-linked genes without a functional W gametolog showed elevated nonsynonymous divergence compared with genes with a W copy (i.e. positive selection-driven faster-Z). However, they concluded that this pattern was attributed to conservation and selective patterns of essential genes rather than an effect driven by selection on hemizygous recessive mutations per se. Genes that had retained their W gametolog were highly dose sensitive, or haploinsufficient (high haploinsufficiency indicates that two copies are necessary for normal gene function; Collins et al. 2022), and broadly expressed across tissues (Xu et al. 2019)—hallmarks of gene essentiality (Bellott et al. 2014, 2017).

Although the efficacy of selection at Y/W gametologs is constrained by reduced effective population size and complete linkage among genes, selection can still shape their evolution. This has been demonstrated in both ancient and recently formed sex chromosome systems (Zhou and Bachtrog 2012b). It is well established that genes with sex-specific functions reside on the Y/W chromosome, such as the male-determining gene *SRY* in mammals (Bellott et al. 2014). Furthermore, functional polymorphisms can sometimes be maintained on the Y/W chromosome through balancing selection acting on traits encoded by Y/W-linked genes (Sandkam et al. 2021; Merondun et al. 2024).

Empirical data on Y/W gene-loss rate, selective dynamics of both gametologs and the role of functional constraint in shaping these processes remain limited (Bachtrog 2008; Huang et al. 2022; Sigeman et al. 2024; Xu et al. 2024). This gap is partly technical, because Y and W chromosomes are often highly repetitive, heterochromatic and structurally complex, making them difficult to assemble and analyse (Tomaszkiewicz et al. 2017). Yet understanding the evolutionary fate of both X/Z- and Y/W-linked genes is essential because sex chromosomes often contribute disproportionately to adaptation, reproductive isolation and life-history evolution (Haldane 1922; Ellegren and Galtier 2016; Meisel 2022). Systems with several, well-defined evolutionary strata are particularly valuable because they allow these processes to be examined across different times since recombination cessation (Bachtrog 2013).

The ancestral avian ZW system originated ∼135 million years ago (MYA), when proto-sex chromosomes carrying the putative male-determining gene *DMRT1* evolved recombination suppression, forming the first stratum now shared by all extant birds (Smith et al. 2009; Wright et al. 2012; Cortez et al. 2014; Zhou et al. 2014). Additional strata later arose independently in different lineages. In passerines (Passeriformes), four ancestral strata are recognized (formed 135–40 MYA; Xu et al. 2019; Zhou et al. 2014). Recent molecular and cytogenetic work have revealed multiple lineage-specific autosome–sex chromosome translocations in birds, generating neo-sex chromosomes followed by renewed recombination suppression and formation of additional strata (Pala et al. 2012; Gunski et al. 2017; Gan et al. 2019; Kretschmer et al. 2020; Burley et al. 2022; Huang et al. 2022; Souza et al. 2024; Xu et al. 2024). These findings challenge earlier assumptions of strong karyotypic stability in birds (Nanda et al. 2008; Ellegren 2010) and instead point to a more dynamic history of avian sex chromosome evolution.

**Fig. 1.**
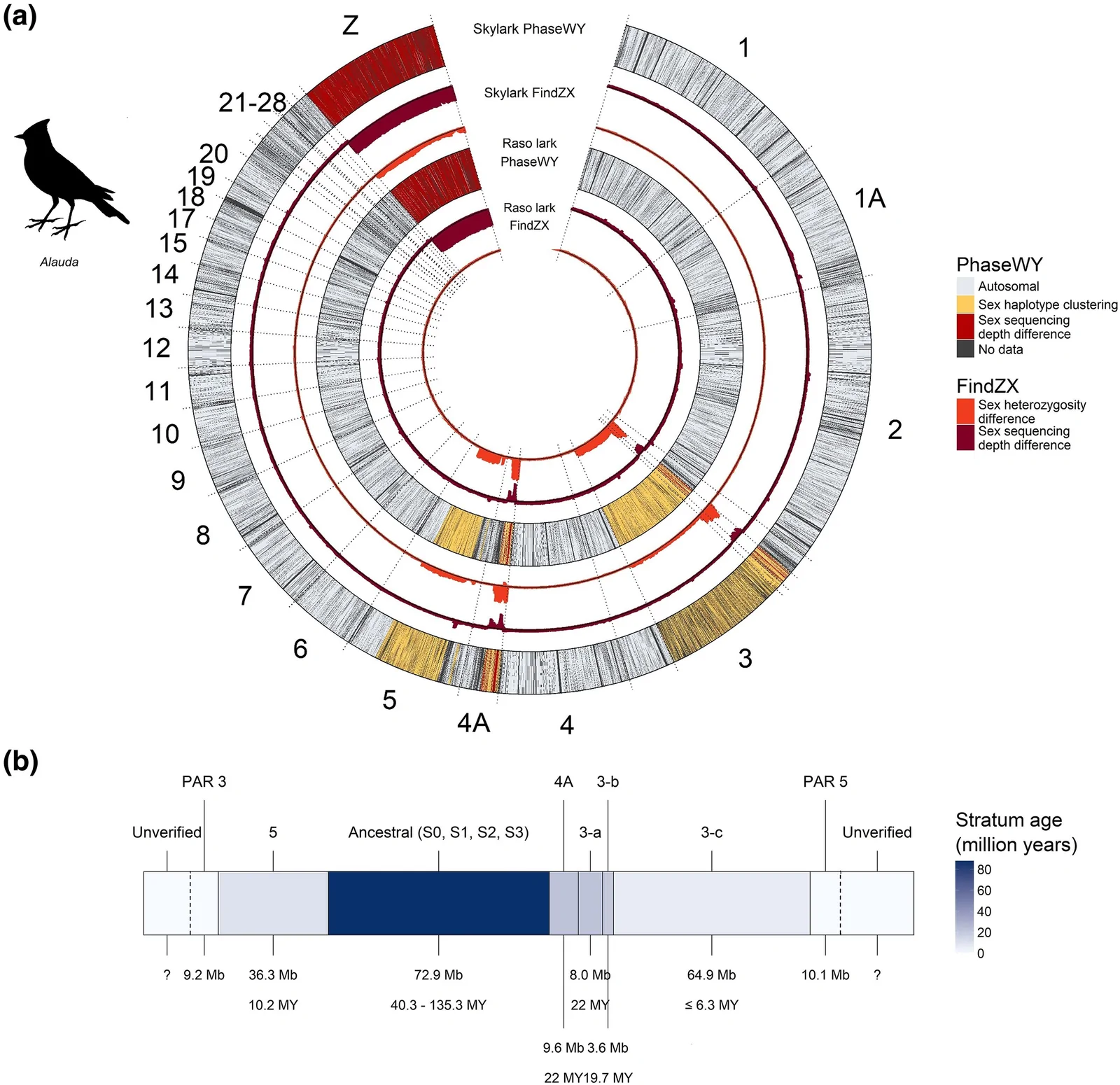
Autosomal and sex-linked regions in the Skylark and Raso lark. a) Genome-wide classification of sex-linkage in the Skylark and Raso lark using two complementary bioinformatic pipelines (*PhaseWY* and *FindZX*), mapped onto the chromosome-level assembly of the Great tit. *PhaseWY* classifications are shown for autosomal regions, sex-linked regions supported by either Z/W haplotype clustering or sex-specific differences in sequencing depth, as well as for regions with missing or unreliable data (including noncallable regions, repetitive regions, uncertain phasing, and unsuccessful synteny alignment). Female–male contrasts from *FindZX* are shown for heterozygosity differences and for sequencing-depth differences. b) Hypothetical structure of the lark Z chromosome, evolutionary strata and PARs. Each stratum is color coded by age, with darker shades indicating older strata. Ancestral strata (S0–S3) are not separated due to complex deep-lineage rearrangements. Parts of the PARs included in this study are indicated (PAR3; PAR5), as are unvalidated parts (Unverified).

Larks (Alaudidae, Passeriformes) exhibit the most extensive of these neo-sex chromosome systems. Three successive translocations involving parts of chromosomes 4A, 3 and 5 expanded the ancestral Z into a large 5–Z–4A–3 neo-sex chromosome (Brooke et al. 2010a; Dierickx et al. 2020; Sigeman et al. 2021, 2019; Fig. 1a). In *Alauda* larks, five additional strata formed subsequently (from ∼22 to ≤6 MYA; Fig. 1b), together producing a 195.3 Mb nonrecombining region corresponding to roughly one-sixth of the ∼1.2 Gb genome (Sigeman et al. 2024, 2019; this study). Karyotypic studies are consistently show large, minimally heteromorphic Z and W chromosomes and fewer macrochromosomes than in other passerines (Bulatova et al. 1971; Bulatova 1973; Roy and Kaul 1985; Sultana and Bhunya 1986; Bian et al. 1988; Malinovskaya et al. 2022). These data suggest that the translocations of chromosome 4A, 3 and 5 encompass substantial chromosome segments and that full sex chromosomes are likely larger than currently defined nonrecombining region. This interpretation is supported by a recent chromosome-level assembly showing that the *Alauda* Z chromosome spans 240 Mb (SJ Ellerstrand et al., unpublished). The extensive and differently aged strata in larks therefore provide an exceptional opportunity to study how sex-linked genes evolve across a broad temporal range following recombination cessation.

Here, we use whole-genome sequence data from the Skylark (*Alauda arvensis*) and the Raso lark (*A. razae*) to investigate the evolutionary dynamics and selection regimes acting on Z–W gametolog pairs across their enlarged neo-sex chromosomes. We investigate how these processes are modulated by stratum age and gene-specific properties, particular gene length and haploinsufficiency. We focus on processes acting after recombination cessation rather than on the initial cause of recombination suppression. Specifically, we aim to (i) identify factors associated with functional loss of W gametologs across strata of different ages; (ii) test whether Z-linked genes show altered divergence after W loss, as expected if hemizygous exposure changes the efficacy of selection; (iii) characterize selective dynamics on W-linked genes and assess whether these covary with selection on their Z gametologs; and (iv) identify Z–W gametologs with signatures of positive selection and evaluate their biological functions to test whether some W genes retain adaptive roles despite recombination cessation. Because Skylarks and Raso larks differ markedly in geographic range and effective population size (Dierickx et al. 2020), we also assess whether recent demographic history modulates signatures of selection across sex-linked regions. By integrating phylogenetic, population-genomic and statistical approaches, this study provides a comprehensive framework for understanding the tempo and mode of sex chromosome evolution across a wide range of stratum ages.

## Results

### Classification of Sex-linked Regions and Extraction of Gametologs

We mapped short-read data from Skylarks (10 females, 8 males) and Raso larks (9 females, 9 males) to a scaffold-level Skylark assembly (scaffold N50 = 1.4 Mb, 5,714 scaffolds, 1.03 Gb; Dierickx et al. 2020). Genomic regions were classified as autosomal or sex-linked, and homologous Z/W-sequences were extracted using two complementary pipelines: *PhaseWY* (https://github.com/sjellerstrand/PhaseWY) and *FindZX* (Sigeman et al. 2021). In Fig. 1a, we show the corresponding autosomal and sex-linked regions of Skylark and Raso lark on the coordinates of the Great tit (*Parus major*) chromosome-level reference genome (GCF_001522545.3; Laine et al. 2016).

Phased sequences were successfully classified and extracted as Z, W or autosomal without evidence of strong sex-biased allele frequencies (Figs. S1 to S3). Raso lark showed almost no sex-biased allele frequency variation, whereas the Skylark Z chromosome displayed minor residual bias, reflected in elevated female variation (Fig. S1). Across the assembly, 83.9% of the bases were callable (excluding repetitive and poorly aligned regions), and 66.9% of the genome was classified as autosomal. Classified sex-linked regions constituted 14.1% of the genome in the Skylark and 16.5% in the Raso lark (Table S3), which was also reflected by a higher number of sex-linked genes retrieved in the Raso lark (1,995) than in Skylark (1,862)—particularly in the youngest stratum, 3-c (Table S4). This difference between species likely arose because the Skylark is substantially more heterozygous, making accurate classification of sex-linked genes more challenging. In total, we extracted protein-coding sequences for 14,195 orthologous genes shared by both species, of which 1,759 were sex-linked and 987 retained full-length W copies (Table S4). From the PARs on chromosomes 3 and 5 (PAR3 and PAR5), 61 and 111 protein-coding sequences were extracted respectively. All gene sequences were based on the Skylark assembly (Dierickx et al. 2020) with annotations from the Zebra finch (*Taeniopygia guttata*) reference genome (GCF_003957565.2; Rhie et al. 2021).

Sex-linked regions overlapped almost completely between species, except for a ∼40 kb segment at the PAR3 boundary of stratum 3-c containing the protein-coding gene *PTP4A1*, which appears rearrangement in Raso lark relative to the Skylark assembly (Fig. S4). Because our analyses required identical gene set across species, *PTP4A1* was excluded from downstream analyses.

### Age of Evolutionary Strata

Evolutionary strata span a wide temporal range across the lark neo-sex chromosomes (Fig. 1b and Table S2). On the ancestral part, strata S0–S3 date to approximately 135 to 40 MY (S0: 135 MYA, S1: 92 MYA, S2: 78 MYA, and S3: 40 MYA). In contrast, the five strata on the added regions are considerably younger, ranging from ∼22 to ≤6 MY (4A: 22 MYA, 3-a: 22 MYA, 3-b: 20 MYA, 5: 10 MYA, and 3-c: ≤ 6 MYA). We estimated generation times decreasing over evolutionary history from 5.3 years/generation in the ancestor of all extant birds to 2.7 years/generation in the Skylark. Thus, stratum ages range from approximately 2.3 to 32.3 million generations (MG; Figs. S5 and S6; Table S2), reflecting a more than 10-fold difference in time since recombination cessation across the lark neo-sex chromosome system.

### Functional Degeneration Across Genomic Regions and Species

**Fig. 2.**
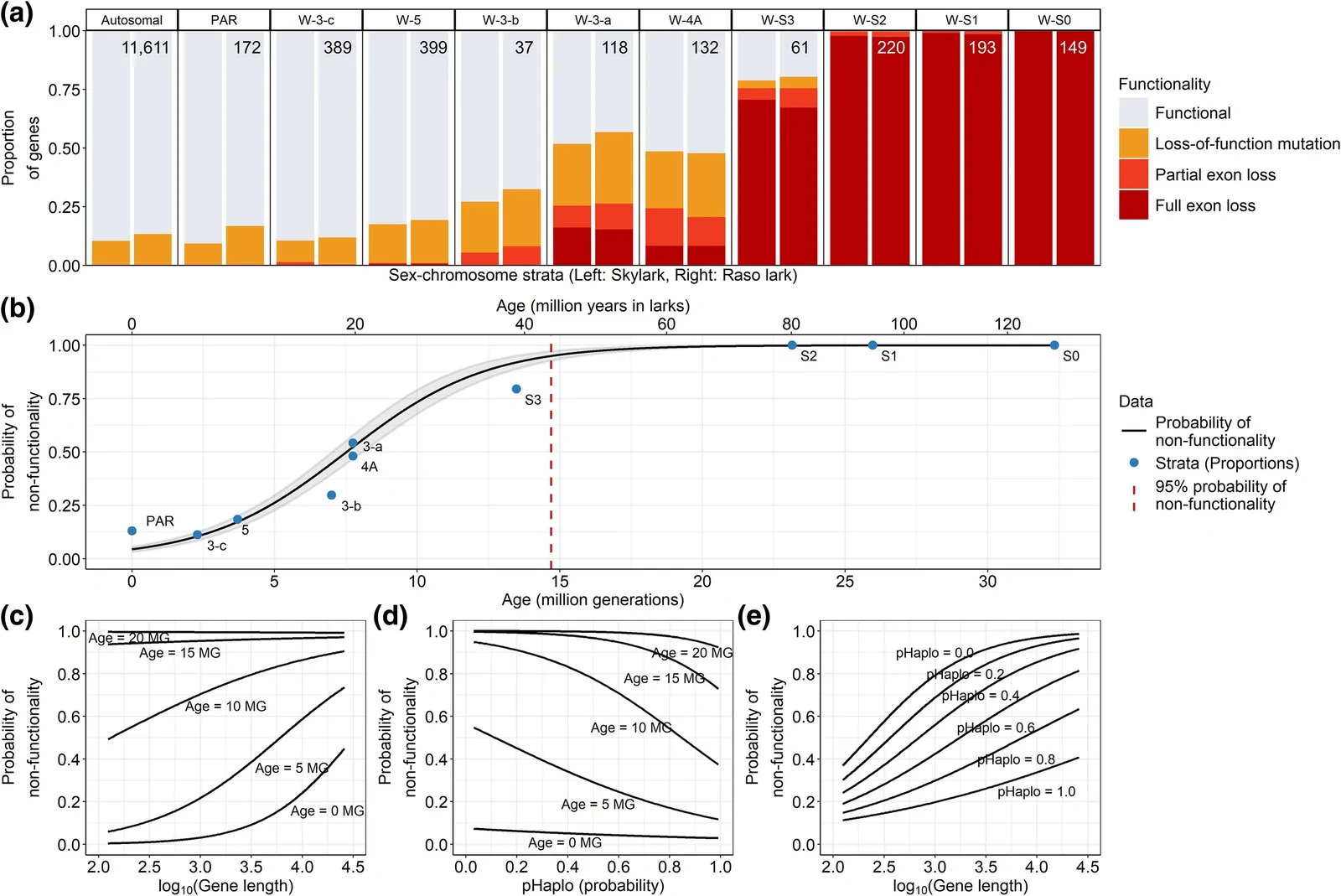
Rate and predictors of W functional degeneration. a) Proportion of genes classified as functional and nonfunctional on autosomes, PAR and W strata in Skylark and Raso lark. Sample sizes (total number of genes and gametolog pairs) are shown for each region. b) Predicted probability of nonfunctionality (±95% confidence intervals) as a function of stratum age (in million generations, MG), with other predictors held at their median values. The equivalent age in years is shown on top axis. Points represent the observed proportion of nonfunctional genes on each stratum (and PAR 3 and 5 combined at age zero). The dashed line indicates the estimated age at which W gametologs reach 95% probability of nonfunctionality. c) Predicted probability of W-gene nonfunctionality as a function of gene length across strata of different ages (MG), with other predictors held at their median values. d) Predicted probability of W-gametolog nonfunctionality as a function of haploinsufficiency (pHaplo) across strata of different ages (MG), with other predictors held at their median values. e) Predicted probability of W-gene nonfunctionality as a function of gene length across various values of pHaplo with strata age set at 7.5 MG.

To quantify functional degeneration across genomic regions (autosomes, PARs and the Z- and W-haplotype of each strata; Table S5) and between species, we classified each gene as functional or nonfunctional. Genes were considered nonfunctional if they (i) carried *SnpEff*-annotated loss-of-function mutations (Cingolani et al. 2012) or (ii) showed partial or complete exon loss inferred from sex-specific sequencing-depth differences (Fig. 2a and Fig. S7). The proportion of nonfunctional genes differed between genomic regions (*P* < 2.0 × 10^−16^) and the Raso lark exhibited more nonfunctional genes than the Skylark across the full genome (log_10_OR = 0.079, *P* = 7.0 × 10^−13^), but the final binomial model (Tjur's *R*^2^ = 0.24) did not include an interaction between species and genomic regions. Post-hoc tests (Bonferroni-corrected) showed that W had a significantly higher proportion of nonfunctional genes than Z in all strata except the youngest stratum 3-c (Fig. S7; Table S5). Unexpectedly, stratum 3-c exhibited a lower proportion of nonfunctional Z-linked genes than the autosomal group (Table S5). No significant differences were detected among autosomes, PAR3 and PAR5 (Table S5).

### Predictors of W Degeneration

As expected, younger strata retained more W-linked genes than older strata, yet several W gametologs persisted for long periods in strata of intermediate age (Fig. 2a). To identify factors explaining functional loss of W gametologs, we modeled the probability of W nonfunctionality as a function of stratum age (million generations, MG), species (Skylark vs. Raso lark), haploinsufficiency (using pHaplo, an index of haploinsufficiency derived from human data; Collins et al. 2022) and gene length (log_10_-transformed), and their interactions. PAR genes were included, representing stratum age zero. We averaged the top five models, all of which showed high explanatory power (Tjur's *R*^2^ = 0.66 for all models; Fig. 2b–e, Fig. S8; Table S6). Effect sizes were calculated as the log_10_ of odds ratios (log_10_OR; Table S6).

The probability of W-gene nonfunctionality increased significantly with stratum age (log_10_OR = 0.42, *P* < 2.0 × 10^−16^) and gene length (log_10_OR = 1.3, *P* < 2.0 × 10^−16^). Several interaction terms were also significant: gene length × stratum age (log_10_OR = −0.056, *P* = 0.0082), haploinsufficiency × stratum age (log_10_OR = −0.11, *P* = 1.1 × 10^−4^), and gene length × haploinsufficiency (log_10_OR = −0.58; *P* = 0.042; Fig. 2b–e, Fig. S8; Table S6). Overall, W genes with high haploinsufficiency were less likely to become nonfunctional (Fig. 2d and e). Species identity had no measurable effect, showing that Skylarks and Raso larks had similar degeneration trajectories. Using median predictor values and averaging across species, we estimated that a typical W gametolog reaches a 95% probability of being nonfunctional after ∼14.7 MG, equivalent to 43.5 MY in passerines/larks (Fig. 2b). Stratum ages and proportions of degeneration were visualized together with estimates from other taxa presented in Krasovec et al., (2018) (Fig. S9).

### Signatures of Selection on Z Gametologs

We inferred signatures of purifying and positive selection acting on Z gametologs using the ratio dN/(dN + dS), based on maximum likelihood estimates of synonymous (dS) and nonsynonymous (dN) substitution rates since divergence from the Great tit. This approach assumes equal total evolutionary time for all genes (i.e. time since the split from the Great tit), but different proportions of that time spent in a sex-linked state depending on stratum age. Genes located in the PAR were included as representing the condition prior to recombination suppression and acting as a proxy for autosomal genes. A substantial proportion of ancestral Z genes (407 of 533) had to be excluded due to few or no detectable substitutions, as dN/(dS + dN) is undefined or unreliable when dS + dN is zero or very small. Such cases were significantly more frequent among ancestral Z genes than among PAR genes (19 of 153; *P* = < 2.2 × 10^−16^; Pearson's Chi-squared test with Yates’ continuity correction).

**Fig. 3.**
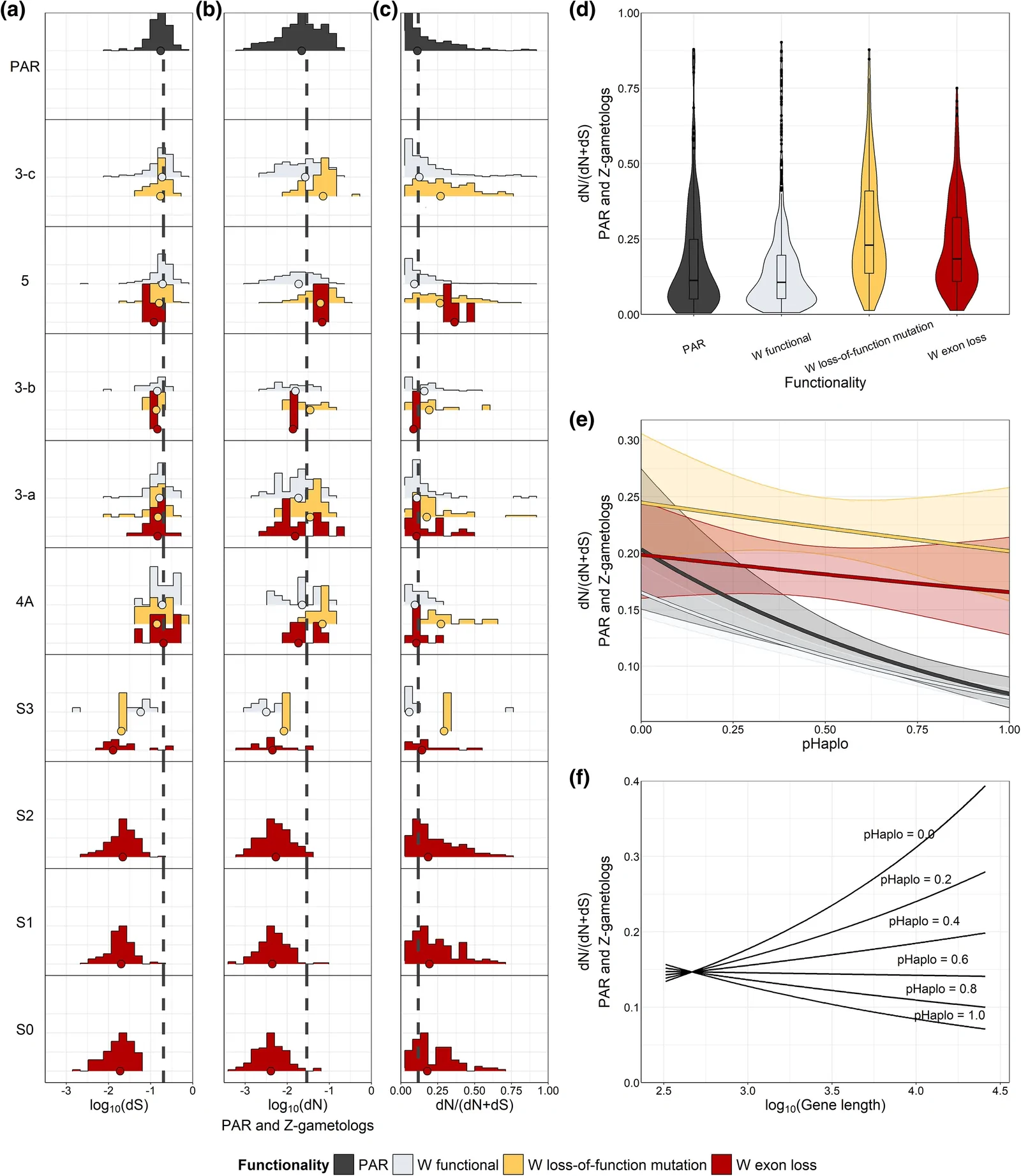
Signatures of selection on Z gametologs. a) Synonymous substitution rate (log_10_(dS)), b) nonsynonymous substitution rate (log_10_(dN)), and c) strength of selection (dN/(dN + dS)) since divergence from the Great tit on PAR genes and Z gametologs grouped by the functional-degeneration status of their W counterpart and arranged by stratum youngest (top) to oldest (bottom). Colored points represent group medians; the median value for PAR genes is shown as a dashed line across all strata. d) Distribution of dN/(dN + dS) for PAR genes and Z gametologs grouped by W functional-degeneration category. e) Estimated interaction between W degeneration and haploinsufficiency (pHaplo), with other predictors set to their median values (±95% CI). Purifying selection strengthens (lower dN/(dN + dS)) with increasing haploinsufficiency (high pHaplo), but this relationship is weaker for Z genes whose W copy is degenerated. f) Signatures of selection (dN/(dN + dS)) of Z and PAR genes as a function of gene length and haploinsufficiency (pHaplo).

Figure 3a–c show the variation in dS, dN and dN/(dN + dS) of Z genes across strata and across classes of W functional status: Z genes with (i) a functional W gametolog, (ii) a W copy harboring loss-of-function mutations, and (iii) a W copy with exon loss (at least one exon lacking from the sequence). To approximate normality, dS and dN were log_10_-transformed, whereas untransformed values were used for dN/(dN + dS). Across the entire Z chromosome (Fig. 3d), signatures of purifying selection clearly dominated (dN/(dN + dS) < 0.5). However, several Z gametologs in intermediate-age strata and paired with degenerated W copies showed relaxed selection (dN/(dN + dS) ≈ 0.5), and a small subset of genes exhibited signatures consistent with positive selection (dN/(dN + dS) > 0.5) (Fig. 3c and d). Overall, Z genes without a functional W gametolog showed higher dN/(dN + dS) relative to PAR genes and Z genes with a functional W copy (Fig. 3d)—indicating a positive selection-driven faster-Z effect.

To explore predictors of Z-selection signatures, we modeled dN/(dN + dS) as a function of W functional status (functional, loss-of-function mutation, exon loss; with PAR genes included as reference), species (Skylark vs. Raso lark), haploinsufficiency (pHaplo), gene length (log_10_-transformed) and their interactions using linear models. Stratum age was excluded due to co-linearity with W degeneration. The top three models were averaged (*R*^2^ = 0.17 for all models; adjusted *R*^2^ = 0.16 for all models; Table S7a) and effect sizes are reported as standardized coefficients (*β*). Significant predictors included gene length (*β* = 0.064, *P* = 0.014) and haploinsufficiency (*β* = −0.30, *P* = 0.010), along with two interaction terms: W functionality × haploinsufficiency (functional: *β* = 0.047, *P* = 0.51; loss-of-function mutation: *β* = 0.23, *P* = 0.0079; exon loss: *β* = 0.24, *P* = 0.0057) and gene length × haploinsufficiency (*β* = −0.087, *P* = 1.3 × 10^−4^; Fig. 3e–f, Fig. S10).

Visualizing these associations (with other predictors fixed at median values and species averaged) revealed two main patterns. First, stronger purifying selection (lower dN/(dN + dS)) was associated with haploinsufficiency for PAR genes and for Z gametologs with a functional W copy, and—but to much less extent—for Z gametologs paired with degenerated W gametologs (loss-of-function mutations or exon loss; Fig. 3e). Secondly, gene length interacted with haploinsufficiency: under high haploinsufficiency, longer genes were under stronger purifying selection, whereas the opposite pattern appeared under low haploinsufficiency (Fig. 3f). These patterns suggest that gene essentiality is an important factor for interpreting differences in selection regimes of PAR and Z genes, and that also long, essential gene can be conserved despite being a large mutational target.

To further dissect our model and evaluate specific processes related to faster-Z theory, we extracted parameter estimates from the model and conducted six additional analyses. (i) We extracted contrasts in dN/(dN + dS) between PAR genes and all Z genes. We found an overall trend suggesting that the rate of nonsynonymous evolution is faster on the Z chromosome (*P* = 0.069). (ii) We extracted the correlation between dN/(dN + dS) and haploinsufficiency (pHaplo) for each of the four gene categories (PAR genes; Z genes with a functional W; Z genes with a W loss-of-function mutation; Z genes with W exon loss). We found that the strength of purifying selection increased (dN/(dN + dS) decreased) with increasing haploinsufficiency for PAR genes (*P* = 1.7 × 10^−6^) and for Z genes with functional W (*P* = 2.5 × 10^−14^), whereas no correlation was detected for Z genes with degenerated W (loss-of-function mutation: *P* = 0.36; exon loss: *P* = 0.33; Table S7b). (iii) We extracted contrasts in dN/(dN + dS) between combinations of gene categories (e.g. PAR genes vs. all Z genes) at five different levels of haploinsufficiency (pHaplo = 0, 0.25, 0.50, 0.75, 1). We found that PAR genes exhibited significantly stronger purifying selection (lower dN/(dN + dS)) than all Z-linked genes at high levels of pHaplo (≥ 0.75; *P* ≤ 0.0038), and likewise when compared only to Z genes with degenerated W for pHaplo ≥ 0.25 (*P* ≤ 0.021). Moreover, Z genes with functional W consistently showed stronger signature of purifying selection (lower dN/(dN + dS)) than Z genes with degenerated W across all five pHaplo levels (*P* ≤ 0.0056; Table S7c). (iv) We compared the Direction of Selection (DoS) between all pairs of gene categories. Z genes with W loss-of-function mutation had the highest DoS (0.054), followed by Z genes with W exon loss (0.037), PAR genes (0.024) and Z genes with a functional W (0.018). Z genes with W loss-of-function mutation and W exon loss were both significantly higher than Z genes with a functional W (loss-of-function mutation: *P* = 2.9 × 10^−4^; exon loss: *P* = 0.0033; pairwise Wilcoxon rank sum test with Bonferroni correction; Fig. S11, Table S8). (v) We compared degree of haploinsufficiency between all pairs of gene categories. PAR genes had the highest pHaplo, followed by Z genes with a functional W, Z genes with W loss-of-function mutation, and Z genes with W exon loss; all pairwise comparisons were significant (*P* ≤ 0.0020; pairwise Wilcoxon rank sum test with Bonferroni correction) except between the two categories of Z genes with degenerated W (Fig. S12, Table S9). (vi) We tested the relationship between log_10_(dN/(dN + dS)) in the autosomal outgroup branch (representing the divergence of the Great tit relative to Zebra finch and Collared flycatcher), and haploinsufficiency (pHaplo) for each of the four gene categories. We found that the strength of purifying selection increased (i.e. dN/(dN + dS) decreased) with increasing haploinsufficiency (*P* = 0.18 × 10^−5^; ANOVA). However, there was no interaction between pHaplo and gene category, and pairwise tests showed that only PAR genes and Z genes with functional W differed significantly (*P* = 0.020; Tukey post-hoc test; Fig. S13, Table S10).

Together, these results support a faster-Z effect in which hemizygous exposure of recessive mutations in females accelerates nonsynonymous divergence at Z-linked genes lacking a functional W copy, a pattern seen across all levels of haploinsufficiency. Importantly, the results do not support a drift-driven faster-Z effect. Under increased drift, we would have expected consistently stronger purifying selection in PAR genes compared with all Z-linked genes regardless of haploinsufficiency, a pattern which we do not observe. Instead, by evaluating rates in combination with polymorphism data (DoS), we found that Z genes with hemizygous exposure show distinctly higher signs of positive selection. Moreover, the selection regime acting on Z-linked genes appears to be strongly shaped by gene essentiality: haploinsufficient gametologs are preferentially retained on the W chromosome, and haploinsufficient Z-linked genes with a functional W copy exhibit particularly strong signatures of purifying selection, comparable to those of PAR genes and highly distinct from Z-linked genes with a degenerated W.

### Signatures of Selection on W Gametologs

**Fig. 4.**
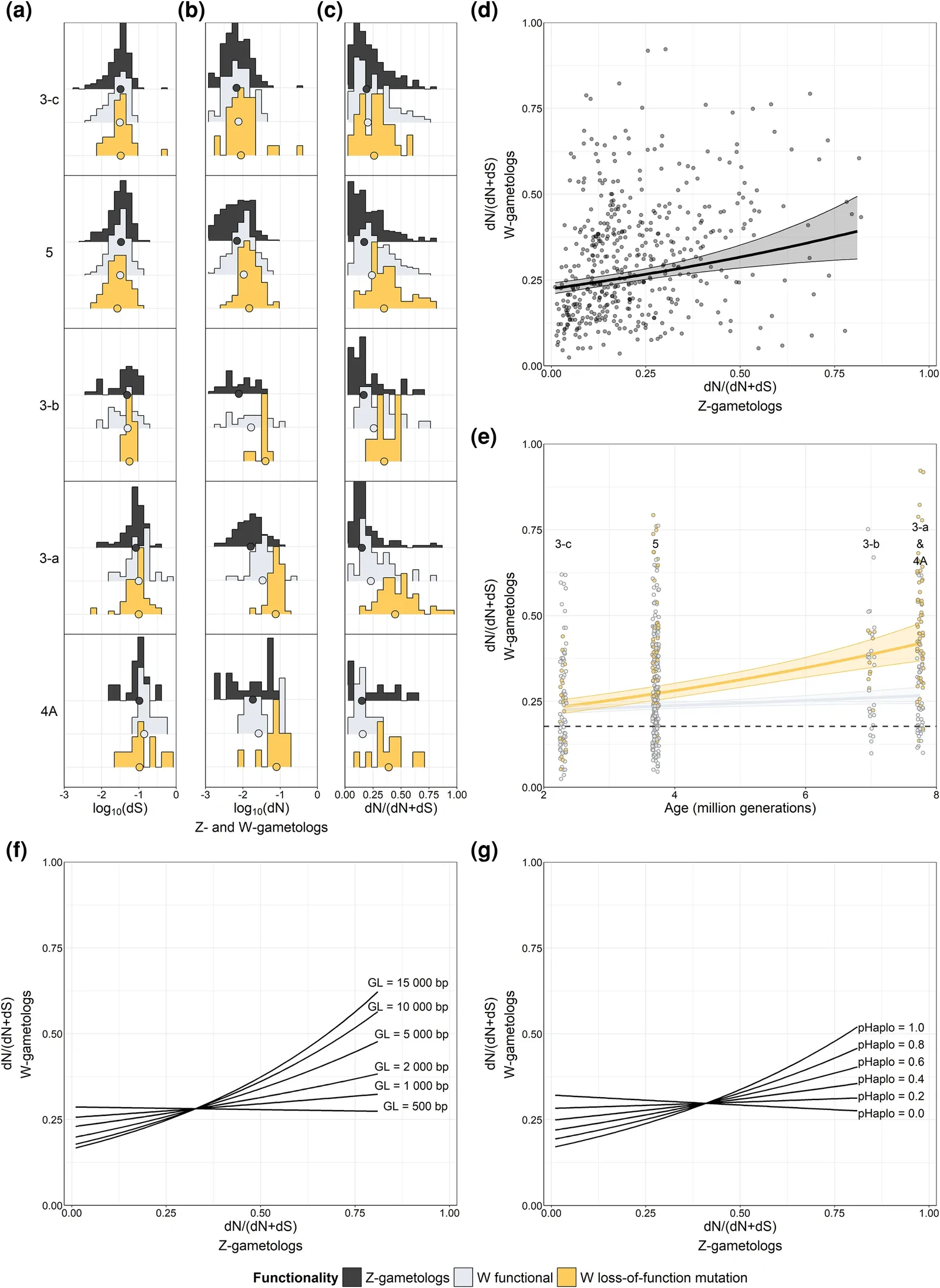
Signatures of selection on W gametologs. a) Synonymous substitution rate (log_10_(dS)), b) nonsynonymous substitution rate (log_10_(dN)), and c) strength of selection (dN/(dN + dS)) since recombination cessation for Z gametologs, functional W gametologs and W gametologs carrying loss-of-function mutation, shown across strata arranged from youngest (top) to oldest (bottom). Colored points indicate group medians. d) Relationship between dN/(dN + dS) of W and Z gametologs (±95% CI); raw data shown as transparent points. e) Interaction between stratum age and W functional status (functional or loss-of-function) on W-selection signature (dN/(dN + dS); ±95% CI). Individual data points are transparent and jittered horizontally for visibility. The dashed line indicates the median Z-selection strength. f) Relationship between dN/(dN + dS) of W and Z gametologs at different gene lengths. g) Relationship between dN/(dN + dS) of W and Z gametologs at different pHaplo.

We analysed signatures of selection on W gametologs following recombination cessation, measures as the ratio dN/(dN + dS). These analyses included functional W gametologs and those carrying loss-of-function mutations (genes with exon loss were excluded due to missing sequence data), and were restricted to genes located on the neo-sex strata (there were too few W genes remaining on the ancestral region). Figure 4a–c show dS, dN and dN/(dN + dS) for functional W genes, W genes with loss-of-function mutations, and their corresponding Z gametologs across strata. To approximate normality, dS and dN were log_10_-transformed, but untransformed values were used for dN/(dN + dS). Overall, purifying selection was consistently weaker on W gametologs (dN/(dN + dS) < 0.5) compared with their Z counterparts (dN/(dN + dS) << 0.5). Notably, a small number of W genes—including some that remain functional—showed signatures consistent with positive selection (dN/(dN + dS) > 0.5; Fig. 4c).

To identify predictors of W-selection signatures, we modeled dN/(dN + dS) as a function of the Z-selection signature, species (Skylark vs. Raso lark), haploinsufficiency (pHaplo), gene length (log_10_-transformed), stratum age (MG), W functional status (functional vs. loss-of-function) and their interactions using linear models. The top 11 models were averaged (*R*^2^ = 0.25 to 0.26; adjusted *R*^2^ = 0.23 to 0.24; Table S11). Significant predictors included Z selection strength (*β* = 0.20, *P* = 0.030), gene length (*β* = −0.061, *P* = 0.023), haploinsufficiency (*β* = −0.11, *P* = 0.013), and several interaction terms: stratum age × W functionality (*β* = 0.25, *P* = 0.0037), Z selection strength × gene length (*β* = 0.095, *P* = 0.041) and Z selection strength × haploinsufficiency (*β* = 0.12, *P* = 0.0078; Fig. 4d–g, Fig. S14; Table S11).

Visualizing these associations (with other predictors fixed at median values and species averaged), revealed three main patterns. First, W-selection signature was positively correlated with Z-selection signature, although W copies overall exhibited weaker signs of purifying selection than their Z gametologs (Fig. 4d). Second, W gametologs carrying loss-of-function mutations evolved under more relaxed selection—dN/(dN + dS) values approaching 0.5—compared with functionally maintained W genes, particularly in older neo-sex chromosomal strata (Fig. 4e). Finally, the correspondence between W- and Z-selection signature increased with gene length (Fig. 4f) and with increasing haploinsufficiency (Fig. 4g).

### Branch-Length Tests for Selection on Gametolog Pairs

**Fig. 5.**
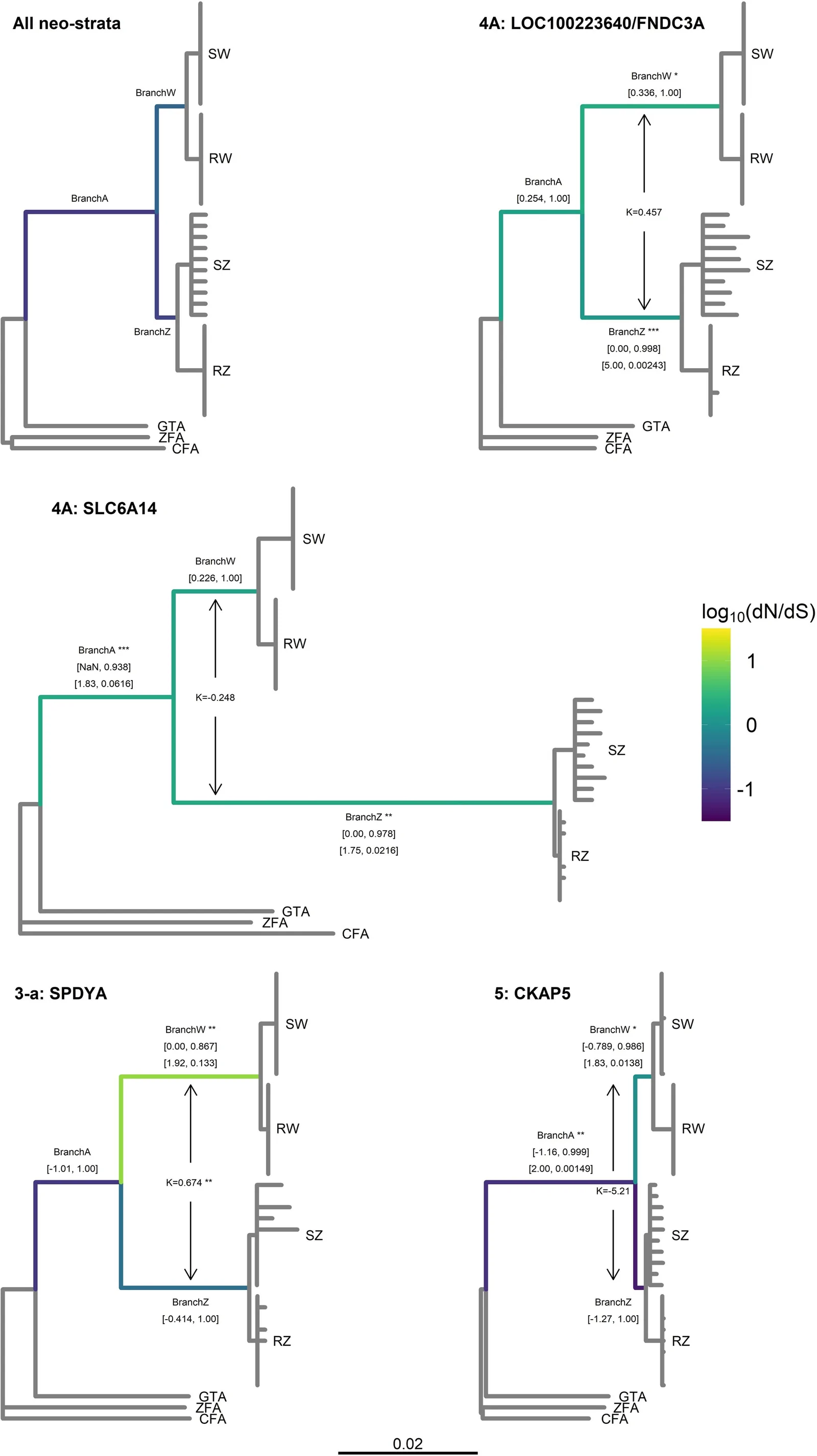
Gene trees and results from branch-length tests for selection on gametologs. Maximum-likelihood estimates of branch lengths and branch-specific dN/dS (Omega; heatmap) were obtained using the MG94 model, with all rates shown as log_10_(dN/dS). Branch length and rate estimates are displayed for (i) all genes on all neo-sex chromosome strata, and (ii) for four specific gametologous pairs. Genes showing significant positive selection (dN/dS > 1) on any branch are annotated with the log_10_ of their aBSREL-estimated rate distributions (values in square brackets). Gametolog pairs showing significant relaxed/intensified selection relative to each other are annotated with the log_10_ K parameter from RELAX (negative values: relaxed W/intensified Z; positive values: relaxed Z/intensified W). Branch labels: SW, Skylark W; RW, Raso lark W; SZ, Skylark Z; RZ, Raso lark Z; GTA, Great tit autosomal; ZFA, Zebra finch autosomal; CFA, Collared flycatcher autosomal. Tips correspond to Z and W sequences of individual females.

Using a phylogenetic framework, we estimated branch lengths and branch-specific rates of sequence evolution using dN/dS (Omega) for 824 gametolog pairs. We also calculated mean branch lengths and average branch-specific dN/dS (weighted by gene length) per neo-stratum and for all neo-strata. Across neo-sex chromosome strata, we observed a consistent pattern of purifying selection (dN/dS < 1) acting on the autosomal branch (prior to recombination cessation) and the Z branch, with slightly relaxed selection on the W branch (Fig. 5, top left). At stratum level, stratum 3-b showed evidence of positive selection (dN/dS > 1) on the Skylark W branch (Fig. S15). We further noted pronounced within-population sequence variation in Skylark Z copies, while Raso lark Z copies—and all W copies—showed comparatively little variation (Fig. S15).

To evaluate selection acting on genes located on neo-sex chromosome strata, we conducted two complementary branch-specific tests. First, we applied the adaptive branch-site random-effects likelihood method (*aBSREL*) to detect positive selection (dN/dS > 1) on three branches: (i–ii) the two gametolog-specific branches after recombination cessation and prior to divergence of Skylark and Raso lark (branch Z and W), and (iii) the autosomal branch preceding recombination cessation, spanning the period from the Great tit divergence to the recombination-cessation event (branch A; Fig. 5). Second, we applied the *RELAX* method to test whether selection on one gametologous branch was relaxed (dN/dS ≈ 1) or intensified (dN/dS < 1 or > 1) relative to its counterpart (i.e. comparing branch W against branch Z).

In total, we identified 40 genes under positive selection on the autosomal, Z or W branch, and 29 genes showing relaxed or intensified selection on Z or W (66 unique genes overall; Tables S12 and S13). Eight W gametologs were under positive selection following recombination cessation (*BRWD3*, *CKAP5*, *FAM161B*, LOC100223640/*FNDC3A*, *OGFRL1*, *SMOC1*, *SPDYA*, *STEEP*), but this gene set did not show enrichment for GO or KEGG terms. We also detected six W gametologs with intensified selection relative to their Z gametologs (*ALKBH1*, *NEK9*, *PRSS35*, *SPDYA*, *WDR43*, *WDR44*), which were enriched for the human KEGG terms “Progesterone-mediated oocyte maturation” and “Oocyte meiosis”. Notably, *SPDYA*, which showed both significant positive selection on the W branch and relaxed Z/intensified W selection, was associated with several human GO terms related to female reproduction, including “oogenesis” and “female gamete generation” (Table S14). We identified four Z gametologs under significant positive selection (LOC100223640/*FNDC3A*, *SLC6A14*, *TBXT*, *URB2*), but this gene set was not enriched for GO or KEGG terms linked to sex determination or sexual differentiation.

Finally, six genes were significant in multiple tests—either in both *RELAX* and one branch in *aBSREL*, or for two branches in *aBSREL*: LOC100223640/*FNDC3A* (stratum 4A; positive Z and W selection), *SLC6A14* (stratum 4A; positive Z and autosomal selection), *SPDYA* (stratum 3-a; positive W and relaxed Z/intensified W selection), *CKAP5* (stratum 5; positive W and autosomal selection), *C5H11orf16* (stratum 5; positive autosomal and relaxed W/intensified Z selection), and *URB2* (stratum 3-c; positive Z and relaxed W/intensified Z selection). Rates for four of these six genes, and for all genes on all neo-sex chromosome strata, are illustrated in Fig. 5.

## Discussion

### Classification of Sex-linked Regions and Extraction of Gametologs

Our classification of sex-linked regions in the Skylark and Raso lark showed strong agreement between *PhaseWY* and *FindZX* and was consistent with previous results from the lark neo-sex chromosomes (Sigeman et al. 2019). With the exception of a small 40 kb rearranged segment at the PAR boundary of stratum 3-c in Raso lark, both species shared the same sex-linked architecture. Phasing and extraction of gametologs with *PhaseWY* opens up several interesting analyses including studies of sex chromosome evolution (Jayaprasad et al. 2024), variation within haplotypes (Almeida et al. 2021) and gametolog divergence (this study).

Using *PhaseWY*, we recovered a substantial and largely overlapping set of gametologs from both species. The slightly lower number retrieved from the youngest stratum in Skylark likely reflects its markedly higher diversity (Dierickx et al. 2020), which reduces the contrast between Z and W haplotypes in recently recombination-suppressed regions. Despite this, phasing success remained high and yielded a shared core dataset of 1,759 gametolog pairs for subsequent analyses. This comprehensive dataset provides a robust foundation for comparative analyses across strata and species, and it demonstrates that even highly polymorphic genomes can deliver well-resolved sex-linked haplotypes when analysed with modern phasing approaches.

### W Degeneration Over Time and Across Strata

Across the neo-sex chromosome, W gametologs showed clear signatures of progressive degeneration in both lark species, consistent with patterns described in other birds (Huang et al. 2022; Sigeman et al. 2024; Xu et al. 2024). All strata except the youngest (3-c) exhibited substantially higher proportions of nonfunctional W copies than their Z homologs or autosomal background, reflecting the cumulative effects of mutation, drift and selection following recombination cessation (Bachtrog 2008). The W decay was strongly stratified: young strata partly retained functionality, whereas the oldest stratum (S0) had lost all W-linked genes. Although the severely bottlenecked Raso lark displayed a slightly higher rate of W-gene loss (53.2%) than in the widely distributed Skylark (51.9%), this effect was uniform across the genome and did not translate into stratum-specific differences. This suggests that most W degeneration pre-dated the species split, and that recent demographic history affects only the residual fraction of still functional genes.

Our statistical models reinforced that stratum age is a dominant predictor of W nonfunctionality. The relationship was nonlinear: degeneration was limited in very young, accelerated rapidly in strata of intermediate age and slowed again in the oldest strata. This pattern likely reflects mutation limitation in young strata and maintenance of a few essential genes in older strata. From these models, we estimated that a stratum typically requires ∼14.7 MG (43.5 MY) to reach 95% nonfunctionality. This rate of degeneration appears slower than estimates from other avian species, but faster than estimates from human, *Drosophila miranda*, and plant XY systems (Krasovec et al. 2018). We also found that gene length strongly influenced W decay, with longer genes degenerating earlier. This suggests that long genes present a larger mutational target and are harder to maintain under reduced efficacy of selection (Ellegren 2011), but they may also be more susceptible to technical artifacts such as alignment and variant-calling errors. The effect of gene length weakened in older strata, where nearly all genes have crossed the threshold to nonfunctionality. By contrast, haploinsufficiency (implying that two functional copies are required for normal function) acted as a stabilizing force: essential, dosage-sensitive genes were more likely to remain functional regardless of their age and length. This aligns with findings across taxa (e.g. Bellott et al. 2017, 2014; Xu et al. 2019; Peichel et al. 2020; Sigeman et al. 2024, 2021) showing that essentiality underpins long-term retention of Y/W genes.

Our results highlight that although recombination suppression initiates a predictable degenerative trajectory, individual W genes can persist for tens of millions of generations. This longevity suggests continued functional contributions of W gametologs to female-specific functions long after the cessation of recombination. However, because our classification focuses on coding changes, it likely underestimates the earliest stages of decay; regulatory erosion, as documented in other systems (*D. miranda*; Zhou and Bachtrog, 2012a), may precede coding degeneration and begin shortly after recombination ceases.

### Signatures of Selection on Z Gametologs

We found that the signature of Z-selection was primarily shaped by W loss and intrinsic gene properties, notably gene length and haploinsufficiency. Z genes whose W gametolog was degenerated (loss-of-function mutation or exon loss) showed elevated nonsynonymous divergence rates (higher dN/(dN + dS)) compared with PAR genes and Z genes with a functional W (Fig. 3d), consistent with a faster-Z effect. Long Z gametologs tended to experience relaxed purifying selection, whereas haploinsufficient genes were markedly more conserved. These effects interacted: haploinsufficiency mitigated the relaxed purifying selection associated with long genes, and haploinsufficient PAR genes and Z genes with a functional W experienced the strongest purifying selection overall. Z genes with a degenerated W copy were on average less haploinsufficient.

That Z genes with degenerated W gametologs consistently exhibited higher nonsynonymous divergence contradicts expectations under hemizygous purging of deleterious mutations, which predicts stronger purifying selection on exposed Z alleles in females. Instead, the pattern is consistent with a positive selection-driven faster-Z effect, whereby hemizygosity increases the efficacy of selection on recessive beneficial mutations. Supporting this interpretation, Z genes with a degenerated W showed higher nonsynonymous divergence than those with a retained W regardless of haploinsufficiency (Fig. 3d). The same pattern was found when incorporating polymorphism data with DoS to compare groups. To our knowledge, this is the first avian study explicitly comparing Z genes with retained versus degenerated W counterparts and finding signatures of consistent with positive selection-driven faster-Z evolution. Wanders et al. (2024) reported stronger faster-Z effect in role-revered, polyandrous shorebirds, although they acknowledge that this pattern may reflect either positive selection-driven faster-Z or more efficient selection of sexual antagonistic genes. Similarly, Dean et al. (2015) found evidence for positive selection-driven faster-Z evolution in Galloanserae based on gene expression patterns.

While evidence for positive selection-driven faster-Z in birds remains limited, they are well-documented in Lepidoptera (Sackton et al. 2014; Mongue et al. 2022). Such effects are thought to be facilitated by large population sizes of butterfly populations, which counteract drift despite the reduced effective population size of Z chromosomes. Consistent with this, Skylarks show high genetic diversity and signatures of long-term large effective population sizes (Dierickx et al. 2020), conditions that may promote positive selection-driven faster-Z evolution. Such demographic scenario may characterize the evolutionary history of the lark family, which has undergone a relatively rapid species radiation (Alström et al. 2013, 2023), although the island-endemic Raso lark has a recent history of small population sizes (Dierickx et al. 2020). Direct estimates of sexual selection in larks are limited. Skylarks and Raso larks are predominantly socially monogamous, although extra-pair paternity has been reported in Skylarks, associated with longer wings—potentially linked to the extended song-flight displays of the males (Hutchinson and Griffith 2008; Brooke et al. 2010b). Furthermore, larks generally display weak sexual plumage dichromatism, while males are typically larger than females (Alström et al. 2013). Thus, the influence of drift on faster-Z evolution may have been limited in larks due to large effective population sizes and relatively modest levels of male sexual selection.

It is notable that we found little evidence for drift-driven faster-Z evolution, which has been proposed as the primary mechanism in ZW systems, including in birds (Mank et al. 2010a; Wright et al. 2015; Xu et al. 2019; Hayes et al. 2020; Chase et al. 2025), copepods (Mongue and Baird 2024) and flat worms (Mrnjavac and Vicoso 2025). Differences from earlier avian studies may reflect their focus on ancestral sex chromosomes where most W genes are already lost, and reliance on deep divergence timescales (88 MY; Mank et al. 2010a; Chase et al. 2025). In contrast, our dataset is dominated by Z gametologs with functional W copies (1,000 genes with W, 562 without W) and more recent divergence (∼23 MY). Because Z genes retaining a functional W copy are disproportionately essential in larks, putative signatures of drift-driven faster-Z evolution are likely weakened in our data by a larger representation of genes evolving under strong purifying selection. When we restricted our comparison to PAR genes and Z genes with degenerated W—a dataset more comparable to those in previous studies—we detected relaxed purifying selection on Z, a pattern that could be interpreted as drift-driven faster-Z evolution. However, more detailed examinations reveal inconsistencies with drift-driven faster-Z effects. Specifically, (i) among the least essential genes (i.e. those with low pHaplo), PAR genes and Z genes with degenerated W exhibited similar selection signatures, and (ii) across all levels of gene essentiality, PAR genes and Z genes retaining a functional W also showed similar selection signatures. This leaves other putative explanations for discrepancies between studies, including differences in divergence times, demographic histories, or selection regimes.

An interesting question is which evolutionary processes act on haploinsufficient genes that have nonetheless lost their W gametolog. One might predict that such genes would experience particularly strong purifying selection—and thus show slow divergence—to maintain at least partial expression in females. Contrary to this expectation, we found that haploinsufficient Z genes with a degenerated W copy showed selection signatures similar to those of less haploinsufficient Z genes with a degenerated W, but higher nonsynonymous divergence than haploinsufficient Z genes with a functional W (Fig. 3e). These patterns could arise from two nonexclusive scenarios: (i) mismatches in haploinsufficiency between humans and larks, allowing some putatively haploinsufficient Z genes to diverge more rapidly than expected, or (ii) relaxation of evolutionary constraints once the W copy is lost. The former implies that unmeasured gene-specific properties predispose certain genes to both rapid Z divergence and W degeneration, whereas the latter is consistent with a positive selection-driven faster-Z effect, whereby recessive beneficial mutations exposed in hemizygous females drive accelerated nonsynonymous divergence, potentially reflecting adaptive change or neo-functionalization of female-specific functions.

A caveat in our study is that most ancestral Z genes in our dataset (407 of 533) show no or very few detectable substitutions over ∼23 MY since the divergence from the Great tit were therefore excluded from the analyses (because dN/(dS + dN) is undefined when dS + dN is zero). This extreme conservation likely reflects long-term purifying selection against recessive deleterious mutations, partly driven by hemizygous selection on Z gametologs following widespread loss of their W counterpart; indeed, 98.5% of these genes lack a functional W gametolog. These highly conserved genes are also far more prevalent than those excluded from the PAR for the same reason. As a result, these ancestral genes may represent a broad class of slower-Z evolution. This interpretation is consistent with theoretical expectations of hemizygous purging (Charlesworth et al. 1987; Vicoso and Charlesworth 2009) and empirical findings in Lepidoptera (Mongue et al. 2022), where ancestral Z genes are similarly highly conserved and faster-Z evolution is largely restricted to younger strata (see also Rousselle et al. 2016). Because our analyses necessarily exclude many of these highly conserved ancestral Z genes, the inferred signal of positive selection-driven faster-Z evolution in younger strata may slightly overestimate divergence across the Z chromosome as a whole. Nevertheless, our results highlights that Z-linked divergence is shaped by multiple interacting factors, including evolutionary time, hemizygous purging and gene-specific properties. Accounting for these factors is essential when comparing faster-Z dynamics across systems or among strata of different ages.

### Signatures of Selection on W Gametologs

The selection signatures of W genes largely mirrored those on their Z counterpart: gametologs experiencing strong purifying selection on the Z chromosome tended to show stronger constraints on the W as well. However, W gametologs consistently evolved under more relaxed purifying selection, and this relaxation was more pronounced once the W copy had lost its function. The progressive relaxation of purifying selection among older, non-functional W genes is expected, and thus provides an internal validation of our loss-of-function annotations.

As for the Z analyses, haploinsufficiency emerged as a key predictor of W-selection signatures. Haploinsufficient W genes retained stronger purifying selection, underscoring the importance of dosage-sensitive functions, or more generally gene essentiality, also on the nonrecombining chromosome. Moreover, the correlation between Z and W selection was strongest for haploinsufficient gametologs, suggesting coordinated evolutionary trajectories for essential genes. Gene length also contributed to W selection dynamics, though differently than for Z genes. Long W genes experienced stronger purifying selection, while short ones evolved in a more stochastic manner; corroborating previous results (Lipman et al. 2002; Soni and Eyre-Walker 2022). An interaction between gene length and Z-selection strength on W-selection signatures further revealed that long gametolog pairs tended to evolve in parallel—either are both highly conserved or both accumulating mutations—whereas short genes showed little correspondence between Z and W.

Together, these findings suggest that the fate of W-linked genes is shaped not only by degeneration and drift but also by underlying gene properties that govern how effectively purifying selection maintains genes in the absence of recombination. Essential and longer genes both show coordinated evolutionary responses between Z and W, while short or nonessential genes drift more freely, accelerating W decay. Note that we also found that longer genes were more likely to lose their W gametolog, and effect that was mitigated by gene essentiality (see above).

### Branch-specific Selection Regimes

Branch-length tests across 824 gametolog pairs confirmed that purifying selection dominates (dN/dS << 1) on both the autosomal branch (prior to recombination cessation) and the Z branch, whereas purifying selection is weaker on the W branch (dN/dS < 1). Only a small minority of genes (31 genes) showed evidence of positive selection (dN/dS > 1), and among these, signatures of positive selection were detected in only a few cases on the W (eight genes) and Z branches (four genes). This pattern is consistent with theoretical expectations that selection on Z- and especially W-linked gametologs becomes less effective once recombination ceases, owing to reduced effective population sizes and/or strong linkage (Hill and Robertson 1966; Smith and Haigh 1974; Charlesworth et al. 1993; Ellegren 2011). At first glance, the relatively small number of positively selected Z genes appears to contrast our conclusion that positively selection-driven faster-Z evolution is present. However, the branch-length statistic aims to detect selection on a proportion of the gene, and is therefore not directly comparable to our other analyses, applying average values of dN and dS across entire genes. Consistent with positively selection-driven faster-Z evolution, three out of the four Z genes showing signatures of positive selection in the branch-length test have lost their W gametolog. These results suggest that pronounced positive selection of Z genes is concentrated to a limited set of genes, while most Z genes experience either relatively weak positive selection or purifying selection.

Tests directly comparing the selective regimes between gametologous gene pairs identified a modest number of cases (23 genes) in which the W gametolog evolved under relaxed purifying selection relative to its Z counterpart. This pattern is again consistent with reduced efficacy of selection on W genes following recombination suppression. However, statistical power is inherently limited for young strata and short genes, as insufficient time has elapsed for mutations to arise and fix in either gametolog. Conversely, many older W genes have already been completely lost due to degeneration and are therefore absent from our analyses, biasing our dataset toward genes located on younger neo-sex chromosome strata. Interestingly, six genes showed intensified selection on the W relative to the Z, and these genes were enriched for pathways related to oocyte maturation and meiosis, suggesting selection acting on female-specific functions.

Finally, six genes were significant across multiple tests, underscoring that individual gametolog pairs can follow highly idiosyncratic evolutionary trajectories. For four of these genes, we found documented roles in reproductive processes across diverse taxa, suggesting that conserved or co-opted functions may be maintained on the W chromosome. These genes were *LOC100223640/FNDC3A* (mouse, dairy cow, blue mussel; Obholz et al. 2006; Gallardi et al. 2021; Göcz et al. 2022; Daudon et al. 2024)*, SLC6A14* (human; Karunakaran et al. 2011; Noveski et al. 2014; Siasi and Aleyasin 2016), *SPDYA* (Chinese mitten crab, hermaphroditic scallop; Hidalgo-Cabrera et al. 2022; Chen et al. 2024), and *CKAP5* (human, mouse; Hu et al. 2025). The remaining two genes (*C5H11orf16/C11orf16* and *URB2*) lacked obvious sex-related functions, suggesting that deviations from general trends in sex chromosome evolution (e.g. purifying selection or degeneration) do not necessarily reflect sex-specific selection.

### Conclusion

In this study, we used the enlarged neo-sex chromosomes of *Alauda* larks to investigate the early and intermediate stages of W degeneration and the selective regimes acting on Z–W gametologs. Across strata, W degeneration followed a predictable trajectory shaped by time since recombination cessation and gene properties, with long genes being lost earliest and essential (haploinsufficient) genes being preferentially maintained. Surprisingly, we did not observe convincing evidence of drift-driven faster-Z evolution, while we found support for selection-driven faster-Z evolution acting through intensified positive selection on hemizygous genes. Our results are one of the few supporting evidence for a positive selection-driven faster-Z effect in birds. However, this effect was modified by gene essentiality: haploinsufficiency intensified the strength of purifying selection on Z while retaining their W gametolog. The signature of W-selection closely mirrored the signature of Z gametologs, with essential genes showing parallel evolutionary trajectories and nonessential genes drifting toward functional loss. Branch-length tests revealed evidence of positive or intensified evolution in a handful of W gametologs, including *CKAP5, FNDC3A* and *SPDYA*, suggesting retained or specialized female-specific functions. Despite pronounced difference in demographic history between Skylark and Raso lark, their sex chromosome evolutionary patterns were remarkably similar, underscoring that most degenerative and selective processes inferred from substitution rates operate over deep evolutionary timescales. Together, our findings highlight the dynamic nature of Z and W chromosome evolution: although most W genes ultimately degenerate, essential Z and W gametologs can retain function and continue contributing to female-specific biology for millions of generations after recombination ceases.

## Materials and Methods

A comprehensive and detailed outline of the Material and Methods (including a complete list of programs, versions and references) can be found as Supplementary material (“Supplementary file: Extended Material and Methods”). Here follows a summary:

### Extraction and Sequencing

DNA was extracted from Skylarks (*n* = 9 females and 7 males) and Raso larks (*n* = 8 males and 8 females; Table S1). The extracts were pair-end sequenced (2 × 150 bp) to ∼30× coverage. Further, we include one female-male pair of each species from Sigeman et al., (2022; Table S1).

### Genome Synteny and Annotation Lift-over

Since the Skylark assembly (GCA_902810485.1) is scaffold level and lacks annotations, we organized the scaffolds according to the chromosome-level Great tit reference genome (*Parus major*; NCBI RefSeq accession GCF_001522545.3; Laine et al. 2016) based on synteny alignment using *Satsuma2* (Grabherr et al. 2010). The scaffolds were annotated for protein coding sequences by lift-over from the well-annotated Zebra finch reference genome (*Taeniopygia guttata*; NCBI RefSeq GCF_003957565.2; Rhie et al. 2021) using *Liftoff* (Shumate and Salzberg 2021). Annotations were also mapped to the assemblies of the Great tit, Collared flycatcher (*Ficedula albicollis*; NCBI RefSeq accession GCF_000247815.1; Kawakami et al. 2014), Ostrich (*Struthio camelus*; Struthio_camelus.20130116.OM.fasta; Yazdi et al. 2023) and Emu (*Dromaius novaehollandiae*; GenBank accession GCF_036370855.1; Rhie et al. 2021). This allowed cross-referencing ancestral Z chromosome strata and associated genes with findings from Xu et al., (2019). Repetitive elements in the Skylark assembly were masked using *RepeatMasker* (Smit et al. 2013-2015).

### Processing of Short-read Data

Sequencing reads were trimmed for quality and adapter removal with *Trimmomatic* (Bolger et al. 2014), and subsequently aligned to the Skylark assembly with *BWA-MEM* (Li 2013 May 26). Duplicate reads were identified and removed. Variant calling was performed separately for each species using *Freebayes* (Garrison and Marth 2012). We filtered raw variants by applying standard filters for low/high depth, low quality, high missingness, mapping bias, improper pairing, strand bias, and excess heterozygosity. Variants located within repetitive elements were excluded. Complex variants were decomposed and normalized into constituent SNPs and INDELs. Only sites that remained polymorphic, or fixed for the alternate allele, were retained for downstream analyses.

### Sex Chromosome Identification and Extraction of W-sequences

We applied our recently developed bioinformatic pipeline *PhaseWY* to the filtered variant datasets. A detailed description and the code for *PhaseWY* is available at GitHub (https://github.com/sjellerstrand/PhaseWY). In brief, *PhaseWY* (i) phases genotypes along scaffolds, (ii) utilizes phased haplotype information to cluster and classify regions as sex-linked or autosomal, and (iii) separates sex-linked sequences into Z or W haplotypes for each individual. It also identifies monomorphic regions as either callable or missing data based on sequencing depth and variant filter thresholds. The output is given in bed- and vcf-files. We analysed the variant datasets on our annotated Skylark scaffolds, while excluding scaffolds lacking annotation. The resulting classification of genome regions was converted to the Great tit genome coordinates using the synteny alignment and the lift-over program *Kraken* (Zamani et al. 2014). To evaluate the classification, we visually compared sex-biased patterns in the datasets using Principal Component Analyses (PCA), the folded Site Frequency Spectrum (fSFS), and the distribution of the Inbreeding Coefficient (F_IS_), both before *PhaseWY* (the full dataset) and after *PhaseWY* (the subset autosomal and Z chromosome datasets) using *Plink* (Purcell et al. 2007) and *VCFtools* (Danecek et al. 2011).

### Gene Sequence Extraction and Alignment

As representative protein coding gene sequence of each gene, we selected the longest transcript that was shared across all species (including outgroups), overlapped callable regions, and was confidently placed either in an autosomal region or a single sex-linked stratum. Coding exons that overlapped degenerated W regions were classified as (i) full exon loss if all coding exons were lost, and (ii) partial exon loss at least one coding exon was lost and at least one remained. For genes with multiple transcript classifications, transcripts with all retained coding exons were prioritized over those with any lost coding exon, and partially lost transcripts were prioritized over completely lost ones.

For each selected coding exon the sequence of each individual and phased haplotype was extracted with *BCFtools* (Danecek et al. 2021) along with the corresponding sequences of the Zebra finch, Great tit and Collared flycatcher. Missing exons were represented as sequences of “N”, and uncallable bases were similarly masked with “N”. The extracted sequences were concatenated into full protein coding transcript sequences using *SeqKit* (Shen et al. 2016). We performed codon-aware sequence alignment using *MACSE* (Ranwez et al. 2018), which allows for stop codons and frameshift mutations, which were later used for the estimation of mutational impact on protein structure. Alignment was performed on all Skylark individuals and all Raso lark individuals separately, and then in a second step to construct a single multispecies alignment.

For analyses of sequence evolution, poorly aligned positions and INDELs were removed with *Gblocks* (Castresana 2000). The alignments were subset to include only female Z and W sequences (i.e. the Z haplotypes of males were excluded), ensuring equal samples sizes between gametologs, as well as one randomly chosen autosomal haplotype per female. For analyses of sequence evolution and selection, we only kept gene sequences with at least 100 codons (300 bp) remaining after quality trimming.

### Identification of Genomic Regions and Age of Evolutionary Strata

We used the bioinformatic pipeline *FindZX* (Sigeman et al. 2022) to identify sex-linked regions based on sex difference in sequence read depth and heterozygosity using synteny to the Great tit as reference. The sex-linked regions were consistent between the Skylark and the Raso lark.

Genes located on the ancestral Z chromosome were categorized into evolutionary strata following the classification of Xu et al., (2019). Transcripts were blasted against each protein databases of Chicken (*Gallus gallus*; NCBI RefSeq accession GCF_000002315.4; Warren et al. 2017) and the Collared flycatcher using *BLAST* (Camacho et al. 2009), then cross referenced to the corresponding stratum in Xu et al., (2019). Genes that could not be matched this way were classified based on the corresponding genomic position identified from the lift-over to the Ostrich and the Emu Z chromosomes.

To improve age estimates of the neo-sex chromosome strata 3-b, 3-c, and 5, we applied the *FindZX* pipeline to single female and male pairs from additional species within the lark family. These data suggest that the translocation of chromosomes 3 and 5 to the sex chromosomes occurred in the ancestor of all larks (SJ Ellerstrand et al. unpubl.; see also Sigeman et al. 2019; Dierickx et al. 2020). However, it remains unclear whether these translocations involved the entire chromosomes. Due to the fragmented nature of the Skylark assembly, we currently lack precise estimates of the size of the PARs in Skylark and Raso lark. Nonetheless, we identified independent evidence of recombination cessation of strata 3-c and 5 to varying degrees across several of the additional lark species (SJ Ellerstrand et al., unpubl.). In two species, the sex-linked regions of chromosomes 3 and 5 extended beyond those identified in the Skylark and Raso lark, suggesting these extended regions are pseudoautosmal. These regions were therefore classified as PARs in the Skylark and Raso lark (PAR3 and PAR5).

The age of strata was inferred based on presence/absence of each stratum across various taxonomic groups (Zhou et al. 2014; Sigeman et al. 2019; Xu et al. 2019; Dierickx et al. 2020), combined with phylogenetic estimates of species divergence times where these strata transitions occur (Table S2; Alström et al. 2023; Cortez et al. 2014; Jarvis et al. 2014; Oliveros et al. 2019; Stiller et al. 2024; Wright et al. 2012; see Supplementary methods “Age of evolutionary strata” and Table S2 for details). Recognizing that evolutionary processes operate over generations rather than calendar years, we scaled the evolutionary rate of birds from years into units of generations. This was achieved by modeling generation times as a phenotypic trait. We used the dated phylogeny from Stiller et al., (2024), which includes 363 bird species representing 218 families, and generation length from Bird et al., (2020). Ancestral generation times were reconstructed using *ape* (Paradis and Schliep 2019), *phytools* (Revell 2024) and *mvBM* (Smaers et al. 2016). Cumulative ages were then calculated in both years and generations. Finally, a polynomial model with a fixed intercept at zero was fitted to scale the age of each evolutionary stratum to its corresponding number of generations.

### Species Variation in PAR Boundary on Stratum 3-c

We investigated the region around the PAR boundary on stratum 3-c on scaffold CADDXX010000137.1 where the Skylark and Raso lark showed differences in the extent of sex-linkage. Pairwise absolute divergence (D_XY_) between the two species for male Z and PAR sequences, and female W sequences, respectively, was calculated using *popgenWindows.py* (https://github.com/simonhmartin/genomics_general). We also investigated linkage disequilibrium (LD) between SNPs using *PopLDdecay* (Zhang et al. 2019) based on female Z- and W-sequences for each species separately.

### Branch Specific Rates of Sequence Evolution

We estimated branch-specific rates of sequence evolution (dS, dN, dN/dS), along their maximum-likelihood estimates (alpha, beta, omega), using *HyPhy* (Kosakovsky Pond et al. 2020) by applying fixed topologies according to gene classification (autosomal or sex chromosomal; ancestral or neo-sex chromosomal) and depending on the presence or absence of the W gametolog in Skylark and Raso lark (fully lost in one, both or neither species). Only transcripts shared across all outgroup species were included in the analysis.

When the full W gametolog is lost in both species, it is not possible to estimate W branch-specific rates of sequence evolution for comparisons with the corresponding Z branch. Therefore, we retrieved sequence evolution along the combined branch from the divergence of Great tit to the tip of the homolog in respective species, enabling absolute comparison of evolutionary rate between autosomal genes and sex-linked Z gametologs from respective evolutionary strata (also when W gametologs are lost). For pairwise comparisons of gametologs where W-gametolog sequences were available, we used the evolutionary rate along the gametolog-specific branches following recombination cessation. Similarly, we retrieved evolutionary rates for genes as autosomal, spanning the branch from the divergence of the Great tit to the point of recombination cessation.

Since all our measurements were calculated along branches from ancestral to derived state (using multiple outgroups), the measurements are polarized, allowing inference of the lineage in which changes occurred. The resulting branch-specific rates and branch lengths were presented on the phylogenetic trees, visualized using the log_10_ of inferred dN/dS. For statistical analyses, we manually calculated the ratio dN/(dN + dS), which helped avoiding divisions by zero in highly conserved genes, and thereby minimizing the exclusion of such genes and reducing potential bias in our statistical analyses. This proved especially important for gametologs on the ancestral Z strata, which are highly conserved, and in which we detected very few substitutions. For estimates of within lineage polymorphisms, we again used *HyPhy* applying fixed topologies as star-trees and calculated global substitution rates. We then incorporated polymorphisms with divergence data to calculate Direction of Selection (DoS) as dN/(dN + dS) - pN/(pN + pS) (Stoletzki and Eyre-Walker 2011).

### Mutational Impact on Open Reading Frames

While a W gametolog can be classified as present or absent based on sex differences in sequence depth, its presence alone does not confirm functionality. We therefore classified W gametologs as functional or nonfunctional based on predicted mutational impacts of called variants on protein models from the Zebra finch using *SnpEff* (Cingolani et al. 2012). Because derived alleles are more likely to be deleterious, we excluded sites fixed for the Zebra finch reference allele, as well as alleles also present in the Great tit or the Collared flycatcher. This filtering ensures that most evaluated alleles are unique to the lineage from the Great tit to the larks and helps reduce artifacts from misalignments. Furthermore, we focussed exclusively on fixed loss-of-function mutations within individual lineages, excluding segregating variants within species. Therefore, each species and autosomal, Z-linked and W-linked lineage was subset and filtered to retain only fixed alternate alleles. Genes were classified as nonfunctional if they carried one or more loss-of-function mutations (i.e. mutations causing loss of start codons, gain or loss of stop codons, or frameshifts; MacArthur et al. 2012).

### Haploinsufficiency

To estimate gene haploinsufficiency, we utilized predicted probability scores (pHaplo) derived from human data, where high scores indicate a higher probability of haploinsufficiency (Collins et al. 2022). These scores were applied to the Zebra finch annotation by cross-referencing one-to-one orthologs between the Zebra finch (bTaeGut1_v1.p) and human (GRCh38.p14) reference genomes accessed through *BioMart* (Kinsella et al. 2011; Ensembl Genes 112). For genes that could not be cross-referenced via gene names, we performed BLAST searches of Zebra finch transcripts against proteins sequences from human (GRCh37.75).

### Branch-length Tests of Selection on Gametolog Pairs

To formally test whether individual gametologs were subject to selection following recombination cessation, we applied the adaptive Branch-Site Random Effects Likelihood (*aBSREL*) method in *HyPhy* using fixed topologies. We only analysed gametologs located on the neo-sex chromosome strata for which full W-gametolog sequences had been extracted from both species. We tested for evidence of positive selection (dN/dS > 1) on each of three specific branches: (i, ii) the W- and Z-specific branches following recombination cessation and prior to the divergence of Skylark and Raso lark (branch W and branch Z), and (iii) the branch preceding recombination cessation, i.e. the branch extending from the divergence with the Great tit to the recombination cessation event (branch A). Holm-Bonferroni correction was applied per gene internally for each test. To assess whether selection on one gametolog had relaxed (dN/dS ≈ 1) or intensified (dN/dS < 1 or >1) relative to its gametolog (i.e. comparing branch W and branch Z), we used *RELAX* from *HyPhy*. Rate distributions were visualized as the log_10_ of inferred dN/dS (omega).

To further investigate the functional roles of genes evolving under different selective regimes, we evaluated GO and KEGG terms. Gene annotations were cross-referenced to chicken and human using *biomaRt* (Durinck et al. 2009). Enrichment analyses were conducted with *clusterProfiler* (Yu et al. 2012) using both chicken and human as reference. For GO enrichment, we focused specifically on terms involved in biological processes, using all protein coding genes across the genome as the background set. We analysed the following gene sets: (i) Z gametologs under significant positive selection, (ii) W gametologs under positive selection, (iii) genes under positive selection preceding recombination cessation, (iv) Z gametologs under relaxed selection relative to W, and (v) all W gametologs under relaxed selection relative to Z.

### Statistical Analyses

All statistical analyses followed a consistent framework, which included (i) evaluation of co-linearity among putative predictors using *performance* (Lüdecke et al. 2021), (ii) model building using *glmmTMB* (Brooks et al. 2017), (iii) model diagnostics using *DHARMa* (Hartig 2022), (iv) model selection and model averaging using *MuMIn* (Bartoń 2024), and (v) post-hoc comparisons using *multcomp* (Hothorn et al. 2008) and *emmeans* (Lenth 2024). Effect sizes were calculated as the log_10_ of odds ratios (log_10_OR) for binomial models, and as standardized coefficients (*β*) for Gaussian models using *effectsize* (Ben-Shachar et al. 2020). Model fit was quantified as the strength of the squared correlation coefficient (either as *R*^2^, adjusted *R*^2^ or Tjur's *R*^2^) using *performance* (Lüdecke et al. 2021).

### Functional Degeneration Across Genomic Regions and Species

We used generalized linear models (GLMs) with binomial error distribution and logit link function to test whether the probability of gene nonfunctionality differed across genomic regions (21 categories, e.g. PAR3, Z stratum 4A and W stratum 5) and between species (Skylark vs. Raso lark). Two-sided post-hoc tests with Bonferroni-corrected *P*-values was used for pairwise comparisons of genomic regions (e.g, W stratum 3-c vs. Z stratum 3-c).

### Predictors of W Degeneration

We used GLMs with binomial error distribution and logit link function to test whether the probability of W-gene nonfunctionality varied with stratum age (million generations, MG), species (Skylark vs. Raso lark), gene haploinsufficiency (pHaplo), gene length (log_10_-transformed) and their interactions. To avoid zero-inflation we excluded all autosomal genes but kept PAR genes as a proxy for a stratum age of zero. We averaged the top models based on AIC (deltaAIC < 2).

### Signatures of Selection on Z Gametologs

We measured the selection regime on Z gametologs as dN/(dN + dS) since the divergence from the Great tit (reflecting equal evolutionary times, but different proportions of time as sex-linked for genes located on different strata). Genes with zero values of dN/(dN + dS) or missing data in any species were excluded from the analysis to avoid zero-inflation. We used linear models (LMs; Gaussian error distribution and identity link function) to investigate the strength of Z selection as a function of W functional status (categorized as functional, loss-of-function mutation or exon loss, with PAR genes included as a reference group), species (Skylark vs. Raso lark), haploinsufficiency (pHaplo), gene length (log_10_-transformed) and their interactions. We excluded strata age and strata identity as predictors due to their strong correlation with the W degeneration categories. We averaged the top models based on AIC (deltaAIC < 2). The best model was used to estimate slopes and test contrasts between W functional categories along the full range of pHaplo scores using *emmeans*.

### Signatures of Selection on W Gametologs

We measured the selection regime on W gametologs as dN/(dN + dS) since recombination cessation of W. The analysis was restricted to genes where the W gametolog was classified as either functional or carrying loss-of-function mutations (i.e. nonfunctional, but with full sequence available). Zero values of dN/(dN + dS) were excluded to avoid zero-inflation. To approximate a normal distribution, the response variable (dN/(dN + dS)) was log_10_-transformed. We used linear models (LMs; Gaussian error distribution and identity link function) to investigate the strength of W selection as a function of strength of Z selection, species (Skylark vs. Raso lark), haploinsufficiency (pHaplo), gene length (log_10_-transformed), strata age (MG), W functionality status (functional or loss-of-function mutations) and their interactions. We averaged the top models based on AIC (deltaAIC < 2).

## Supplementary Material

evag204_Supplementary_Data

## Data Availability

The sequence data used in this study are publicly available on NCBI. Accession IDs are given in Supplementary Table S1. All code and scripts used in this study are provided at GitHub: https://github.com/sjellerstrand/Lark_sex_chrom_genomics.
