# Supplementary material for: Selective Regimes and Evolutionary Dynamics of Z and W Gametologs Across an Expanded Avian Neo-Sex Chromosome
Source: Genome Biol Evol. 2026 Aug 10;18(8):evag204. doi: 10.1093/gbe/evag204 (PMC13524101; doi:10.1093/gbe/evag204)
Supplement: Supplementary file 1 [file evag204_supplementary_data.zip › methods_260803.docx]

Supplementary file: Extended Material and Methods

**Title:**

Selective regimes and evolutionary dynamics of Z and W gametologs across an expanded avian neo-sex chromosome

**Author information:**

Simon J Ellerstrand^1^, Bengt Hansson^1^

^1^Department of Biology, Evolutionary Ecology and Infection Biology, Science for Life Laboratory, Lund University, Ecology Building, 223 62 Lund, Sweden

ORCID:

SJE 0000-0003-2674-6997
BH 0000-0001-6694-8169

Contents

[1. Sample acquisition and sequencing	3](#_Toc231922178)

[2. Bioinformatic processing of data	3](#_Toc231922179)

[2a. Quality trimming	3](#_Toc231922180)

[2c. Repeat regions and consensus reference genomes	3](#_Toc231922181)

[2d. Genome synteny and annotation lift-over	4](#_Toc231922182)

[2e. Alignment to reference genome	4](#_Toc231922183)

[2f. Variant calling and filtering	4](#_Toc231922184)

[2g. Sex chromosome identification and extraction of W-sequences	5](#_Toc231922185)

[2h. Gene sequence extraction	6](#_Toc231922186)

[2i. Codon-aware alignment and trimming	6](#_Toc231922187)

[3. Analyses	7](#_Toc231922188)

[3a. Definition of genomic regions and evolutionary strata	7](#_Toc231922189)

[3b. Species variation in PAR boundary on stratum 3-c	8](#_Toc231922190)

[3c. Age of evolutionary strata	9](#_Toc231922191)

[3d. Branch specific rates of sequence evolution	10](#_Toc231922192)

[3e. Mutational impact on open reading frames	11](#_Toc231922193)

[3f. Haploinsufficiency	12](#_Toc231922194)

[3g. Gene filtering	12](#_Toc231922195)

[3h. Branch-length tests for selection on gametolog pairs	13](#_Toc231922196)

[3i. GO and KEGG enrichment analyses	13](#_Toc231922197)

[3j. Statistical analyses	13](#_Toc231922198)

[3j1. Functional degeneration across genomic regions and species	14](#_Toc231922199)

[3j2. Predictors of W degeneration	14](#_Toc231922200)

[3j3. Signatures of selection on Z gametologs	15](#_Toc231922201)

[3j4. Signatures of selection on W gametologs	15](#_Toc231922202)

[5. References	17](#_Toc231922203)

# 1. Sample acquisition and sequencing

Blood samples from Skylarks were collected by Pavel Zehtindjiev in Italy (2007), and by Arne Hegeman in the Netherlands (2009). DNA from these samples was extracted using a phenol-chloroform-based protocol. Blood samples from Raso lark were collected by Michael de L. Brooke on the islet of Raso, Cape Verde (2015 and 2017; Table S1). DNA from these samples was extracted from dried blood on tissue paper with using the Qiagen blood and tissue kit (Qiagen, Hilden, Germany). We sequenced DNA extracts of nine female and seven male Skylarks, and eight female and eight male Raso larks as paired-end 150 bp reads on an Illumina NovaSeq 6000 platform. Library preparation of the eight Skylarks from the Netherlands included a PCR-amplification step, while all other libraries were PCR-free. Library preparation and sequencing were performed by National Genomics Infrastructure (NGI), Uppsala, Sweden (<http://ugc.igp.uu.se/our-services/ngs-technologies/>). Additional sequences from one male and one female of each species were retained from Sigeman et al. (2019). DNA from these samples had been extracted from blood using a phenol-chloroform protocol and sequenced PCR-free as paired-end 150 bp reads on an Illumina HiSeq X platform (NGI, Uppsala).

# 2. Bioinformatic processing of data

All code is deposited on <https://github.com/sjellerstrand/Lark_sex_chrom_genomics>.

## 2a. Quality trimming

Raw sequences were trimmed for quality and adapters with *Trimmomatic* v.0.36 (Bolger et al. 2014). Bases with Phred score < 20 were trimmed from the leading and trailing ends, and in averages of 4 bases in sliding windows. Reads < 120 bp were excluded post-trimming and only reads with both pairs remaining were kept for further analyses. Both raw and quality trimmed reads were evaluated for quality with *FastQC* v.0.11.8 (<https://www.bioinformatics.babraham.ac.uk/projects/fastqc/>).

## 2c. Repeat regions and consensus reference genomes

We mapped our reads to a Skylark reference genome (GenBank accession GCA_902810485.1). Since the Skylark reference contains ambiguity codes, the reference was modified by choosing one of the representative bases at random with *convert_ambiguity.py* (<https://github.com/markravinet/convert_ambiguity_codes/blob/master/convert_ambiguity.py>, accessed 2021-12-20). All ambiguity codes were converted except “Ns”.

Repetitive elements were identified in the modified reference genome with *RepeatMasker* v.4.1.0 (Smit et al. 2013 2015), using the repeat database “Aves” as the source of query DNA.

To improve mappability to the reference genome, a consensus reference genome was created for each species using the respective male with the highest sequence count. Reads were aligned to the modified Skylark reference, and variants were called and filtered (see methods described for the full datasets below). Specifically, half-calls resulting from allele-specific filtering with *vcflib* v.1.0.1 were re-genotyped to homozygotes with a custom python script *correct_halfcalls.py* (<https://github.com/sjellerstrand/scripts_general/blob/main/correct_halfcalls.py>).

Finally, *BCFtools* v.1.14 (consensus) was used to apply fixed alternate SNPs to the modified Skylark reference genome to create the new consensus reference for each species. Not applying INDELs thus preserve coordinates to the original reference sequence.

## 2d. Genome synteny and annotation lift-over

Since the Skylark reference genome is fragmented and lacks annotations, we utilised genomic resources available from other passerine species. The Great tit (*Parus major*) is the closest related species to larks with a chromosome-level assembly (NCBI RefSeq accession GCF_001522545.3), which has not undergone any of the neo-sex chromosomal translocations observed within Sylvioidea. To classify the ancestral chromosomal origin of Skylark scaffolds, we performed a whole-genome alignment between the Skylark and Great tit assemblies with *Satsuma* v.2.0 (<https://github.com/bioinfologics/satsuma2>; Grabherr et al., 2010).

For a lift-over of genome annotations of protein coding genes, we used the slightly more divergent, but well-annotated Zebra finch assembly (*Taeniopygia guttata*; NCBI RefSeq accession GCF_003957565.2; Rhie et al., 2021; W scaffolds excluded). We used *Liftoff* v.1.6.3 (Shumate and Salzberg 2021) to map annotations from the Zebra finch to the Skylark assembly. Duplicate genes were removed with *cgat* v.0.3.3 (<https://cgat-apps.readthedocs.io/en/latest/CGATInstallation.html>). In addition, annotations were mapped to the Great tit and the Collared flycatcher (*Ficedula albicollis*; NCBI RefSeq accession GCF_000247815.1; Kawakami et al., 2014) for use as outgroups in downstream analyses. Annotations were also mapped to the Ostrich (*Struthio camelus*; Struthio_camelus.20130116.OM.fasta; Yazdi et al., 2023, Table S2) and the Emu (*Dromaius novaehollandiae*; GenBank accession GCF_036370855.1; Rhie et al., 2021, W scaffolds excluded) due to their highly conserved Z chromosome synteny, allowing us to cross-reference ancestral Z chromosome strata and respective genes based on the results by Xu et al. (2019).

## 2e. Alignment to reference genome

Read pairs were aligned to the consensus reference genome with *BWA-MEM* v.0.7.17 (Li 2013 May 26). *MarkDuplicates* from *Picard* v.2.23.4 (<http://broadinstitute.github.io/picard>) was used to identify and remove read duplicates. *SAMtools* v.1.14 (Li et al. 2009) was used to exclude reads that were not mapped in proper pairs, not primarily aligned, or with a mapping quality score < 20 (view -f2 -F260 -q20).

## 2f. Variant calling and filtering

Variants were called with *Freebayes* v.1.3.2 (Garrison and Marth, 2012), excluding alignments with a mapping quality < 30, alleles with a supporting base quality < 20, alignments with a depth > 200 times the number of individuals, and sites with a depth < 3, only evaluating the best 10 SNP alleles.

We filtered raw variants with a combination of *VCFtools* v.0.1.16 (Danecek et al. 2011), *vcffilter* from *vcflib* v.1.0.1 (Garrison et al. 2022), and *BCFtools* v.1.14 (Danecek et al. 2021). We excluded variants within repeat regions, variants with a quality score < 30, variants with a missingness < 0.95, and genotypes with a depth < 5. We further applied species-specific filters, such that the maximum mean variant depth was set was set to 1.5*mean depth (Skylark 55.7, Raso lark 46.4). In very divergent sex chromosome regions where female W-reads do not align to the homologous Z reference, female genotypes will result in about half the mean female individual depth. We therefore set the minimum mean variant depth low by dividing mean female individual depths by two, then set the mean minimum variant depth to a third that of the new mean of all individuals (Skylark 8.7, Raso lark 7.5). Further, we filtered sites with excess heterozygosity (all non-missing genotypes heterozygous).

Filtering of alleles at very low frequencies is usually performed in an attempt to remove single sequencing errors at the cost of filtering out true, rare variants. This is unfortunate, since they are essential for some analyses such as demographic inference (O’Leary et al. 2018). We therefore avoided using filters of minor allele frequencies by instead applying filters inspired by O’Leary et al., (2018). Since these specific filters are originally intended for Restriction site-Associated DNA sequencing (RADseq) data, they were partly modified to apply to whole genome sequencing data. Alternate alleles were excluded if they had a quality < 30 (-f "QA = 30 | QA > 30"), a large discrepancy in mapping quality relative to the reference allele (-f "MQMR = 0 | (( MQM / MQMR ) > 0.25 & ( MQM / MQMR ) < 1.75 )"), if the properly paired status was low or had a large discrepancy relative to the reference allele (-f "PAIREDR = 0 | ( PAIRED > 0.05 & PAIREDR > 0.05 & ( PAIREDR / PAIRED ) < 1.75 & ( PAIREDR / PAIRED ) > 0.25 ) "), or were not supported by at least one read on each strand, and by at least two read centred to the left and right side of the variant (-f "SAF > 0 & SAR > 0 & RPL > 1 & RPR > 1").

*Freebayes* v.1.3.2 often produces haplotype variants and complex alleles. These were decomposed and normalized into their constituent SNPs and INDELs with a combination of programs. We used *vcfallelicprimitives* from *vcflib* v.1.0.1, followed by *decompose_blocksub* from *vt* v.0.5772 (<http://github.com/atks/vt>). Alleles were then normalized with *normalize* from *vt* v.0.5772 (Tan et al. 2015), and duplicate records removed with *BCFtools* v.1.14. Any phased genotypes were set as unphased. Only sites remaining polymorphic or fixed for the alternate allele were kept.

The effects of the filters on the datasets were routinely evaluated visually throughout the filtering process. The distributions of different variant characteristics were evaluated with *VCFtools* v.0.1.16 by site quality, site mean depth, proportion of missing data per site, and minor allele frequency. The distributions of individual-specific traits were also evaluated through the inbreeding coefficient, mean individual depth, and proportion of missing data per individual. Further, the distribution of the allelic balance (the frequency of the minor allele vs. the major allele) at heterozygote genotype calls was evaluated for each individual independently using a slightly modified version of *checkHetsIndvVCF.sh* (<https://github.com/speciationgenomics/scripts/blob/master/checkHetsIndvVCF.sh>).

## 2g. Sex chromosome identification and extraction of W-sequences

We applied our recently developed bioinformatic pipeline *PhaseWY* on our filtered variants to phase genotypes along scaffolds, then used haplotype information to cluster and classify regions as sex-linked or autosomal, and to separate out sex-linked sequences as Z or W in females. *PhaseWY* identifies monomorphic regions of the genome as callable or missing data based on sequencing depth and filter thresholds applied to variants and summarises this information in bed and vcf-files. We ran our datasets on our annotated scaffolds (annotated with coding regions by lift-over from the Zebra finch genome; above), while scaffolds lacking annotated genes were excluded. The resulting classification of sections of the genomes was converted to the coordinates of the Great tit genome, using the previous synteny alignment, and the lift over program *Kraken* (Zamani et al. 2014). The classification was evaluated by visually comparing sex-biased patterns in the datasets with PCAs, the folded Site Frequency Spectrum (fSFS), and the distribution of the Inbreeding Coefficient (F_IS_) on both the full dataset before the data were processed through *PhaseWY*, and the subset autosomal and Z chromosome datasets after *PhaseWY*. *Plink* v.1.90b4.9 (Purcell et al. 2007) was used to linkage-prune the data using 50 kb windows, a step sizes of 10 kb, and an r^2^-value of 0.1, and to calculate the principal components of remaining independent variants. The fSFS and F_IS_ were calculated with *VCFtools* v.0.1.16 (--freq2 and --het). A detailed description and the code for *PhaseWY* is available at GitHub (https://github.com/sjellerstrand/PhaseWY).

## 2h. Gene sequence extraction

Protein coding gene sequences (CDS) resulting from the lift-over were selected for further analyses based on the following set of criteria: (i) only transcripts in the Zebra finch annotation containing both a start and a stop codon were included, (ii) any transcripts with duplicate features were excluded, (iii) features not overlapping callable regions at a minimum of 80% were excluded, (iv) transcripts containing individual CDS features overlapping at least 90% to either autosomal or sex-linked regions were excluded, and (v) transcripts with different CDS features overlapping at least 90% to more than one evolutionary stratum or unknown stratum were excluded. CDS features that overlapped heterogametic-dropout regions by at least 90% were classified as lost on the W chromosome. If any sex-linked transcript had lost all CDS features, it was classified as completely lost. If any sex-linked transcript had lost some but not all CDS features it was classified as partially lost, and remaining CDS features were used to extract a W-sequence. In genes with transcripts of multiple classifications, the transcripts with all retained CDS features were chosen in favour of transcripts with any lost CDS features, and partially lost transcripts were chosen in favour of completely lost transcripts.

For each gene, the longest transcript of the remaining features was chosen for further analyses, provided it was shared with all outgroup species, which was a prerequisite for being used in the analyses of rate of evolution. Transcripts were then chosen with precedence given to transcripts where (i) all CDS features of a transcript had been successfully lifted over from the Zebra finch, and (ii) the start codon having been successfully lifted over and remained in the annotation file following previous filtering of features. The sequence of each CDS feature for each individual and phased haplotype were then extracted with *BCFtools* v.1.14 (*consensus*) together with the corresponding sequence of the Zebra finch, Great tit and Collared flycatcher. Missing exons were extracted as sequences of “N” and uncallable bases were likewise masked with “N”. All sequences were collected in multi-fasta format files, and *SeqKit* v.0.15.0 (Shen et al., 2016; *concat*) was used to concatenate all CDS sequences in order into full protein coding transcript sequences. Only genes with transcripts identified in both the Skylark and the Raso lark were used for further analyses.

## 2i. Codon-aware alignment and trimming

Sequences from complete, protein coding genes of all individuals were codon-aware aligned with *MACSE* v.2.07 (Ranwez et al., 2018; alignSequences) separately for Skylark and Raso lark. *MACSE* accounts for stop codons and frameshift mutations (which was used for the estimation of mutational impact on protein structure; see below). For alignments of genes that included a W gametolog, the W sequences were specified as less reliable (-seq_lr), lowering any penalties for insertions of frameshifts and stop codons. The sequence alignments of the Skylark and Raso lark, respectively, were then aligned in a second step to a single multispecies alignment (*alignTwoProfiles*). These alignments were further processed for different analyses. For estimation of mutational impact on protein structure, leading gaps (“-“) and subsequent frameshift mutations (“!”) relative to the Zebra finch were trimmed. For analyses of sequence evolution, poorly aligned positions and INDELs were identified and removed using *Gblocks* v.0.91b (Castresana 2000). All “!” and “N” in the alignment were transformed to alignment gaps as “-“ to be recognised by *Gblocks*. The type of sequence was set to codon (-t=c). As the alignment contains a majority of identical within-species sequences, the minimum number of sequences to define a conserved position was increased to the total number of sequences per alignment (-b1). We further used “relaxed” parameter settings according to Talavera and Castresana, (2007), setting the minimum number of sequences for a flank position to the same as for a conserved position (-b2), the maximum number of contiguous non-conserved positions to 10, and the minimum length of a block to 5, but not allowing any gap positions (-b3=10 -b4=5 -b5=n). *SeqKit* v.0.15.0 was used to subset the alignments to only female Z- and W-sequences to achieve an even samples size between gametologs, as well as one randomly chosen autosomal sequence from every female.

# 3. Analyses

## 3a. Definition of genomic regions and evolutionary strata

We used the bioinformatic pipeline *FindZX* (Sigeman et al., 2022a, accessed 2021-12-09) to identify sex-linked regions based on sex difference in alignment depth and heterozygosity. This pipeline was previously used to identify sex-linked regions from a single female and male pair for each of the Skylark and Raso lark (Sigeman et al. 2019), and here we apply it to all our samples. We modified *FindZX* to input our already aligned reads, as well as our filtered vcf-files, and used the Great tit as synteny species. *FindZX* outputs sex-linkage along the genome in a continuous format, not converted to a discrete classification. To compare the result with that of *PhaseWY*, we manually classified sex-linked regions from the output. Windows bordering evolutionary strata, or non-sex-linked regions (likely caused by erroneous assembly), were classified as unknown stratum. Two scaffolds were identified as sex-linked regions bordering the PAR (CADDXX010000058.1 on chromosome 5 and CADDXX010000137.1 on chromosome 3, stratum 3-b). All sex-linked regions corresponded between the Skylark and the Raso lark. However, minor adjustments were made due to differences in classification of the extent of the PAR scaffold CADDXX010000137.1 based on the *PhaseWY* pipeline, a region of which was absent from the *FindZX* output. In this region, the sex-linked region extended to include the gene *PTP4A1* in the Skylark, but not in the Raso lark. The species-specific classifications were thus set accordingly.

Due to the fragmented nature of the Skylark assembly, our knowledge of the size of the PARs only extends to these two scaffolds. However, using the *FindZX* pipeline on single female and male pairs of several species of larks to infer presence/absence of sex-linked strata, we found independent evidence of recombination cessation of strata 5 and 3-c in several species with various extents (see “Age of evolutionary strata”). Translocations of chromosome 5 and 3 to the sex chromosomes were present in the ancestor of all larks and likely included large parts or all of chromosome 5 and 3. In two species, we found PARs extending further than that found in the Skylark and Raso lark. These included stratum 5 in *Spizocorys sclateri*, and stratum 3-c in *Plocealauda affinis* (S. J. Ellerstrand et al., unpublished). The sex-linked regions in these two species were therefore further classified as PARs in the Skylark and Raso lark, while any other regions with synteny to chromosomes 5 and 3 were classified as unknown PARs to separate them from the category of regular autosomal regions. Regions with uncertain PAR classification were not used in any further analyses. The automated genome classification from the *PhaseWY* pipeline was intersected with the manual classification based on *FindZX* to exclude regions of unknown sex-linkage, as well as to subset and define regions as callable or uncallable.

Genes located on the ancestral Z chromosome were further organised into evolutionary strata according to Xu et al. (2019). We built two independent BLAST databases based on proteins in Chicken (NCBI RefSeq accession GCF_000002315.4; and the Collared flycatcher using *BLAST* v.2.15.0+ (Camacho et al. 2009). The Zebra finch transcript chosen for each gene (see Gene sequence extraction) was then blasted against each protein database with *blastx*, only retaining e-values < 1e-6. The top hit was matched to respective genome annotation to retrieve gene name and gene ID (Chicken), which were cross referenced to the strata in Xu et al. (2019). Using a similar approach, the original gene name originating from the Zebra finch lift-over was cross-referenced. We retrieved the genomic position of each gene from a lift-over to the Ostrich and Emu. The stratum associated with respective gene was primarily chosen based on the top Chicken BLAST-hit using gene ID, then on Zebra finch gene name match, and finally on the top Collared flycatcher BLAST-hit using gene name match. If none of these methods worked, the stratum was decided based on the corresponding position in the Ostrich and Emu Z chromosomes if the gene had successfully been annotated from respective lift-overs. If none of these criteria were fulfilled, the gene could not be assigned to any stratum and was not in included in further analysis.

## 3b. Species variation in PAR boundary on stratum 3-c

We closely inspected the region around the PAR-boundary on stratum 3-c, located on scaffold CADDXX010000137.1, that showed variation between the Skylark and the Raso lark. We estimated pairwise absolute divergence (D_XY_) along the entire scaffold using *popgenWindows.py* (<https://github.com/simonhmartin/genomics_general>, accessed 2021-12-09). We compared the Skylark and Raso lark Z sequences extracted from males, including both the sex-linked and recombining regions. Because *PhaseWY* is not designed to determine which PAR-haplotype is physically linked to the sex-linked W sequence in females, we compared the Skylark and Raso lark W sequence only in the sex-linked region. As each species had been aligned to its own consensus reference genome, resulting in variation in reference alleles, we (i) extracted the alleles from each haplotype, (ii) created scaffold-wide fasta sequences with *BCFtools* v.1.17 (*consensus*), and (iii) concatenated these into a multi-fasta-file, which was converted into vcf-format with the Skylark consensus genome as reference, using *msa2vcf.py* (<https://github.com/pinbo/msa2snp/blob/master/msa2vcf.py>, accessed 2023-11-14). D_XY_ was calculated in sliding windows (2,000 bp windows with 500 bp steps; based only on callable sites to avoid bias through missing data).

Pairwise linkage disequilibrium (LD) between SNPs was calculated as the square of the correlation coefficient (r^2^) using *PopLDdecay* (Zhang et al., 2019, Accessed 2023-07-16). LD was estimated separately for each species, using only female Z- and W-sequences, across both the sex-linked and recombining regions. Because pairwise comparisons of all SNPs within a scaffold can result in large and redundant datasets, SNPs were subsampled along the scaffold. To account for variation in SNP density between species, and across the scaffold in females (where SNP density is inflated in sex-linked region due to gametologous Z- and W-sequences), we down-sampled each dataset to achieve comparable sample sizes and approximately uniform SNP density. We divided the scaffold into non-overlapping windows of ~10 kb and randomly selected up to 12 SNPs per window. We calculated LD only for SNPs separated by no more than 100 kb. Finally, average LD values were calculated along the scaffold in sliding windows of 5,000 bp in 1,250 bp steps.

## 3c. Age of evolutionary strata

The age of strata were inferred based on their presence or absence in various taxonomic groups (Zhou et al. 2014; Sigeman et al. 2019; Xu et al. 2019; Dierickx et al. 2020), combined with phylogenetic estimates of species divergence times where these patterns occur (Table S2). To refine the age estimates for the neo-sex chromosome strata 3-b, 3-c and 5, we infer presence/absence of strata by applying *FindZX* on single female and male pairs of selected taxa within the lark family (*Alaudala cheleensis*, *Ammomanes cinctura*, *Calendulauda albescens*, *Eremophila alpestris brandti*, *Eremopterix hova*, *Plocealauda affinis*, and *Spizocorys sclateri*). These findings are referred to in the main text (introduction) but will be part of a lark-wide study on variation of recombination cessation (S.J. Ellerstrand et al., unpublished).

For the ancestral stratum S0, we used divergence estimates from studies that have calculated pairwise distances between Z- and W-sequences of four different gametologous genes (Wright et al. 2012; Cortez et al. 2014). These estimates were first averaged within each study, then across studies. For strata S1 and S2, we used the averaged node ages from multiple methods reported by Jarvis et al. (2014; Table S16), which were further averaged with node ages from Stiller et al. (2024). For stratum S3, we used node ages from Oliveros et al. (2019) and Stiller et al. (2024), and averaged these values. The ages of neo-sex chromosomal strata were inferred from dated phylogenies of all birds (Stiller et al. 2024), passerines (Oliveros et al. 2019) and the lark family (Alström et al. 2023). Node ages were averaged across studies to achieve final estimates.

The oldest stratum S0 formed in the ancestor of all today living birds (Neornithes), and was estimated to an age of 135.250 my (133.5-137 my; Cortez et al., 2014; Wright et al., 2012). Stratum S1 formed after Neognathae diverged from Palaeognathae but before the divergence of the Neoaves from Galloanseres (Zhou et al., 2014), placing it at an age of 91.958 my (95.693-88.223 my; Jarvis et al., 2014; Stiller et al., 2024). Stratum S2 formed after Neoaves diverged from Galloanseres, and is present in the Brown mesite (*Mesitornis unicolor*; Zhou et al., 2014), placing it before Passerea diverged from Columbea at an age of 78.112 my (88.223-68.001 my; Jarvis et al., 2014; Stiller et al., 2024). The most recent of the ancestral songbird stratum, S3, appears to have arisen independently in Rifleman (*Acanthisitta chloris*) and the infraorders Passerides and Corvides (Xu et al. 2019), placing it at an age of 40.347 my (48.712-29.987 my; Oliveros et al., 2019; Stiller et al., 2024). The youngest neo-sex chromosomal stratum, 4A, is present in all species of the superfamily Sylvioidea (*sensu* Alström et al., 2006; Fregin et al., 2012) and absent from its closest sister groups (Pala et al. 2012; Sigeman et al. 2019; Sigeman, Zhang, et al. 2022). However, it ceased recombining independently in basal lineages of Sylvioidea, including the lineage of the bearded reedling (*Panurus biarmicus*) and the lark family (Alaudidae) that diverged early from the rest of Sylvioidea (Sigeman et al. 2019; Sigeman et al. 2021; Sigeman, Zhang, et al. 2022), placing it at an age of 21.991 my (23.043-20.938 my; Alström et al., 2023; Oliveros et al., 2019; Stiller et al., 2024). Likewise, stratum 3-a was formed after the bearded reedling (*Panurus biarmicus*) and the lark family (Alaudidae) diverged from other Sylvioidea, but is present in both groups (Sigeman et al. 2019; Sigeman, Zhang, et al. 2022). We do not know whether recombination cessation occurred as an independent event from stratum 4A, placing it at the same age of 21.991 my (23.043-20.938 my; Alström et al., 2023; Oliveros et al., 2019; Stiller et al., 2024). Stratum 3-b was formed after the lark family diverged from the bearded reedling, but is present in all deep lark lineages (Sigeman et al., 2019; unpublished data), placing it at an age of 19.741 my (20.938-18.544 my; Alström et al., 2023; Stiller et al., 2024; unpublished data). Stratum 5 formed after the lark genera *Alauda, Galerida*, *Lullula*, and *Spizocorys* diverged from other larks in the subfamily Alaudinae (Dierickx et al., 2020; Sigeman et al., 2019; unpublished data), placing it at an age of 10.180 my (11.088-9.271 my; Alström et al., 2023). The youngest stratum, 3-c, formed after the genus *Alauda* diverged from *Galerida*, but is polyphyletic within *Alauda*, where it is present in the Raso lark and Skylark, but is polymorphic within the Skylark (Dierickx et al. 2020; Alström et al. 2023). We therefore set the youngest age at the divergence of the Raso lark and the Skylark but acknowledge that it could be younger. This places the stratum at and age of 6.238 my (7.129-5.348 my; Alström et al., 2023).

To scale the evolutionary rate in units of generations rather than years, we modelled ancestral generation times as a phenotypic trait. We used the dated avian phylogeny generated by Stiller et al., (2024), which includes 363 species in 218 families. Larks were represented by a single species, *Alaudala cheleensis*, where we applied the generation time of *A. arvensis*. Each taxon was matched to their corresponding generation length from Bird et al. (2020; Table S4), and these values were inverse transformed to approximate a normal distribution. Ancestral generation times were reconstructed using the R packages *ape* v.5.8 (Paradis and Schliep 2019), *phytools* v.2.3-0 (Revell 2024) and *mvBM* implemented in the *evomap* v.0.0.0.9000 (Smaers et al. 2016). Branch lengths were rescaled using *ace* (method=”REML”) and the multiple variance Brownian motion (mvBM) model. Ancestral generation times were then inferred with *anc.Bayes* using the rescaled tree and 15,000,000 iterations, discarding the first 5,000,000 as burn-in. To trace branch lengths and generation times from node to node backwards in time from the Skylark to the root, we used the *getMommy* function in *RRphylo* v.2.8.1 (Castiglione et al. 2018). Finally, we calculated cumulative age in both years and generations and fitted a polynomial model (with a fixed intercept at 0) to scale the age of each evolutionary stratum to its corresponding number of generations.

## 3d. Branch specific rates of sequence evolution

We estimated branch-specific rates of sequence evolution using *HyPhy* v.2.5.60 (Kosakovsky Pond et al. 2020). The MG94 codon substitution model with a corrected F3x4 estimator was fit to female-only alignments to calculate branch-specific substitution rates (dS, dN, dN/dS), along their maximum-likelihood estimates (alpha, beta, omega). Fixed topologies were provided based on how the gene was classified - whether autosomal or sex chromosomal, ancestral or neo-sex chromosomal - and whether the the W-gametolog sequence was lost (fully lost Skylark, Raso lark, both, or neither). We only included transcripts shared across all outgroup species in the analyses.

When the full W-gametolog is lost in both species, it is not possible to generate W-branch-specific rates of sequence evolution for comparisons with the corresponding Z-branch. In such cases, we retrieved sequence evolution along the combined branch from the divergence point of the Great tit to the tip of the homolog in each species. This allowed an absolute comparison of evolutionary rate between autosomal genes and sex-linked Z-gametologs from respective evolutionary strata. For the ancestral strata, we did not use outgroup W-sequences for the comparison of ancestral W evolution, as this was incompatible with our chosen outgroup species. However, we still estimated evolutionary rate along ancestral Z-strata to enable comparison with autosomal regions. For pairwise comparisons of gametologs where W-gametolog sequences were available, we calculated evolutionary rate along the gametolog-specific branches following recombination cessation. Likewise, we retrieved evolutionary rate of the genes as autosomal, from the divergence with the Great tit to the point of recombination cessation. Because all measurements were calculated along branches from ancestral to derived states using several outgroup species, the results are polarized. This allowed us to infer in which lineage evolutionary changes occurred.

The resulting branch-specific rates and lengths were used to calculate phylogenetic trees, visualised as log_10_(dN/dS) (omega; see “Branch-length tests for selection on gametologous pairs”). For statistical analyses, the branch-specific maximum-likelihood substitution rate estimates were used to calculate a new metric. We divided non-synonymous substitutions by the sum of both synonymous and non-synonymous substitutions (dN/(dN+dS)), which retains more information when few substitutions have occurred (Stoletzki and Eyre-Walker, 2011). By avoiding divisions by zero we could include more highly conserved genes, which could have biased our statistical analyses if excluded. For estimates of within lineage polymorphisms, we again used HyPhy v.2.5.60, applying fixed topologies as star-trees and calculated global substitution rates. We then incorporated polymorphisms with divergence data to calculate Direction of Selection (DoS) as dN/(dN + dS) - pN/(pN + pS) (Stoletzki and Eyre-Walker, 2011).

## 3e. Mutational impact on open reading frames

While the presence (or absence) of a W-gametolog can be inferred from sex differences in sequence depth, its presence alone does not confirm functionality. Therefore, we classified gametologs as functional or non-functional (pseudogenized) based on the predicted mutational impacts on open reading frames. We used *SnpEff* v.4.3t (Cingolani et al. 2012) to annotate the putative functional impacts of called variants, using protein models from the Zebra finch. Only transcripts shared across all outgroup species were included. Gene alignments were converted to vcf-format using the Zebra finch as reference with *msa2vcf.py* (<https://github.com/pinbo/msa2snp/blob/master/msa2vcf.py>, accessed 2023-11-14). Alleles were decomposed with *vcfallelicprimitives* from *vcflib* v.1.0.1, and multiallelic sites were split into biallelic records (norm -m -). Because derived alleles are more likely to be deleterious, and as the Zebra finch reference allele is not evaluated with this method, we excluded sites fixed for the reference allele, as well as any allele that was also segregating in the Great tit or the Collared flycatcher. This ensured that most evaluated alleles were unique to the lineage from the Great tit to the larks and helped reduce artifacts from misalignments. Furthermore, we focused only on fixed loss-of-function mutations within individual lineages, excluding variants currently segregating in populations. Each species and its autosomal, Z-linked, and W-linked lineages were subset and filtered to retain only fixed alternate alleles.

The Zebra finch genome used in this study was not available in our version of *SnpEff*, so we constructed a new database for predicting mutational impacts. This database was built on the single transcripts previously selected for each gene, by filtering the Zebra finch annotation file with *cgat* v.0.3.3 (gtf2gtf --method=filter --filter-method=transcript --map-tsv-file). To estimate pseudogenization, we classified any gene within a lineage as loss-of-function (LOF) if it carried mutations predicting to disrupt protein coding, such as loss of start codons, gain or loss of stop codons, or frameshifts (MacArthur et al., 2012). Genes for which *SnpEff* reported Zebra finch transcripts as containing multiple stop codons were excluded for further analysis.

## 3f. Haploinsufficiency

To estimate gene haploinsufficiency, we used two indexes derived from human data: the percentage haploinsufficiency index (%HI), where low scores indicate higher likelihood of haploinsufficiency (Huang et al., 2010; Haploinsufficiency Predictions Version 3), and the predicted probability of haploinsufficiency index (pHaplo), where higher scores indicate higher probability of haploinsufficiency (Collins et al. 2022). Haploinsufficiency scores were transferred to the Zebra finch annotation by cross-referencing gene names of one-to-one orthologs between Zebra finch (bTaeGut1_v1.p) and human (GRCh38.p14), accessed via BioMart (Kinsella et al., 2011; Ensembl Genes 112). For genes that could not be directly cross-referenced on gene names, we (i) constructed a BLAST database using Human protein sequences (GRCh37.75) with *BLAST* v.2.15.0+, and (ii) blasted the Zebra finch transcript already selected for each gene (see Gene sequence extraction) against this database with *blastx*, and retained gene IDs with e-value < 1e-6. The gene ID for each top hit was matched to the human annotation to retrieve the corresponding gene name, which then could be assigned a haploinsufficiency score. If the gene name could not be cross-referenced to any of the haploinsufficiency scores, the process was repeated for a maximum of 10 top blast hits. If none of these criteria were fulfilled, the gene could not be assigned to any haploinsufficiency scores. Both scores were evaluated in model selection, and pHaplo was chosen as the best representative of haploinsufficiency (See “Statistical analyses”).

## 3g. Gene filtering

Sex-linked genes that could not be confidently classified into an ancestral sex chromosomal stratum, and autosomal genes on chromosomes 3 or 5 that could not be confidently placed in the PAR region, were excluded from further analysis (see “Definition of genomic regions and evolutionary strata”). Additionally, genes for which *SnpEff* reported multiple stop codons in their Zebra finch transcripts were excluded from further analyses (see “Mutational impact on open reading frames”).

We retained only genes with transcripts identified in all species, including the outgroup species (see “Gene sequence extraction”). For analyses of sequence evolution and selection, we further only kept gene sequences with ≥100 codons (≥300 bp) after trimming. For each respective analysis, we only included genes where the branch under investigation had an alpha value > 0.001, and both beta and alpha values < 1, as values > 1 may indicate mutation saturation.

## 3h. Branch-length tests for selection on gametolog pairs

To test whether individual gametologs were under selection following recombination cessation, we used *aBSREL* (adaptive Branch-Site Random Effects Likelihood) from *HyPhy* v.2.5.60, providing fixed topologies. We restricted the analysis to gametologs on neo-sex chromosome strata for which a full W-sequence had been extracted in both species. We tested for evidence of positive selection (dN/dS > 1) on three branches in the topology: (i,ii) the gametolog-specific branches from the point of recombination cessation to the point of divergence of Skylark and Raso lark (branches W and Z), and (iii) the ancestral branch leading from the divergence with the Great tit to the recombination cessation event (branch A). Holm-Bonferroni correction was performed internally for three branches in each test. To test whether selection on one gametolog had relaxed or intensified relative to the other gametolog, we used *RELAX* from *HyPhy* v.2.5.60, also with fixed topologies. Rate distributions were presented as the log_10_ of inferred dN/dS (omega).

## 3i. GO and KEGG enrichment analyses

To assess the function of genes evolving under different selective regimes, we utilised GO and KEGG terms. Using *biomaRt* v2.62.0 (Durinck et al. 2009), we first matched Zebra finch gene names to alternative IDs, and then matched those to orthologous chicken and human IDs. We performed GO and KEGG enrichment analyses using *clusterProfiler* v 4.14.4 (Yu et al. 2012) against both chicken and human. For GO enrichment, we focussed specifically on biological process terms and used all protein-coding genes across the genome as the background set. We analysed the following sets of significant genes: (i) Z-gametologs under positive selection, (ii) W-gametologs under positive selection (iii) all genes under positive selection preceding recombination cessation, (iv) Z-gametologs under relaxed selection relative to W, and (v) W-gametologs under relaxed selection relative to Z.

To further investigate gene function of gametolog pairs evolving under notable dynamics, we focussed on genes where either (i) more than one branch showed positive selection, or (ii) one branch showed positive selection and selection had relaxed/intensified on Z relative to W. These were further investigated through a brief literature search. We searched the Web of Science core collection with the following search string:

<GENE> AND (gamet* OR sperm* OR egg OR oocyte* OR ovari* OR ovulat* OR gonad* OR testi* OR teste* OR genital* OR estrog* OR testost* OR sex* OR reproduct* OR oogene* OR uter* OR androg*OR estrous* OR fertil*)

## 3j. Statistical analyses

As one of our aims was to compare the Skylark and Raso lark, we included only genes with data available from both species in our analyses. To assess co-linearity among all putative predictors, we calculated and evaluated variance inflation factors using *check_collinearity* from *performance* v.0.13.0 (Lüdecke et al. 2021). All models were built using *glmmTMB* v.1.1.10 (Brooks et al. 2017), and model diagnostics – including checks for residual normality and variance homogeneity – were performed with *DHARMa* v.0.4.7 (Hartig 2022). Model selection and averaging were conducted using *MuMIn* v.1.48.4 (Bartoń 2024). Post-hoc comparisons were performed using *glht* from *multcomp* v.1.4-28 (Hothorn et al. 2008) and *emmeans* v.1.10.7 (Lenth 2024). Effect sizes were calculated as log_10_ of odds ratios (log_10_OR) for binomial models, and as standardised coefficients (*β*) for Gaussian models, using *effectsize* v1.0.0 (Ben-Shachar et al. 2020). Model fit was assessed either as *R*^2^, adjusted *R*^2^ or Tjur’s *R*^2^ calculated with *performance*.

### 3j1. Functional degeneration across genomic regions and species

To evaluate the degree of functional degeneration between genomic regions we modelled the probability of gene non-functionality using binomial models with *glmmTMB.* The model included autosomal genes, and Z- and W-gametologs, classified as either functional or non-functional, with non-functional genes being those carrying loss-of-function mutations (based on *SnpEff* analysis) or – specifically for W gametologs – genes that had lost at least one exon (based on sex differences in read sequencing depth). We investigated how long it takes for recombination cessation to significantly change the proportion of functional genes on W as compared to the autosomes, and if there is a similar change on the corresponding region of the Z chromosomes. We initially included genomic regions (categorical variable; 21 categories: autosomal, PAR 3, PAR 5, Z-S0, W-S0, Z-4A, W-4A, etc.), species (categorical variable; 2 categories: Skylark and Raso lark) and their interaction as fixed factors. Since no functional genes were identified for some ancestral W strata (S0, S1 and S2), a dummy functional gene was added per species for each stratum to enable calculation of a *P*-value. Evaluation with *MuMIn* suggested that both factors without the interaction was the best model with a cumulative weight of 1. This model passed diagnostics with *DHARMa*. A two-sided post-hoc test using Bonferroni correction was conducted with *multcomp* to evaluate if selected pairs of genomic regions differed. We tested autosomes vs. each of the other regions, PAR3 vs. PAR5, and each W stratum to its corresponding Z stratum.

### 3j2. Predictors of W degeneration

To describe the degeneration of W gametologs, we excluded all autosomal genes to avoid zero-inflation but retained PAR genes as a proxy for a stratum age of zero. The response variable was set as binomial, where autosomal genes, Z- and W-gametologs were classified either as functional or non-functional. Non-functional genes included those carrying loss-of-function mutations (based on *SnpEff* analysis) or had lost at least one exon (based on sex differences in read sequencing depth). We included predictor variables hypothesised influence W degeneration: (i) strata age (continuous variable: time since recombination cessation in generations), representing the duration a has been exposed to inefficient selection, (ii) species (categorical variable; 2 categories: Skylark and Raso lark), capturing short-term effects of differences in genetic drift, (iii) haploinsufficiency (continuous variable), representing the potential negative impact of having only a single functional gene copy, (iv) gene length (in the Zebra finch; (continuous variable), based on the rational that shorter genes present a smaller mutational target and make them easier to maintain functional. Gene length was log_10_ transformed due to strong positive skew, which otherwise prevented model convergence. Genes with missing data for either haploinsufficiency score (%HI and pHaplo) were excluded. We assessed co-linearity with *performance* and found no signs of co-linearity (highest VIF = 1.24). However, since pHaplo and %HI both measure haploinsufficiency and are highly correlated (*P* < 2.2 × 10^-16^), we did not include them in the same model. We constructed two global models, each incorporating all predictors and second-degree interactions, using either %HI or pHaplo. Model selection was performed with *MuMIn dredge*, ranked by AIC. The top 5 models included pHaplo (and not %HI) and had AIC values ranging from 1889.6 - 1891.5, compared to 1903.0 - 1904.4 for models including %HI. We therefore continued with analyses using pHaplo. The top five models had AIC differences < 2, indicating none were significantly less suitable than the best model. The cumulative model weight was 0.557. We evaluated the residuals of each of the five models individually with. Each model passed the diagnostic checks using *DHARMa* and we averaged them with *MuMIn model.avg*. Effect sizes were calculated as log_10_ odds ratios for each predictor. Functional relationships were visualised by plotting variable while holding all other predictors at their median distributions.

### 3j3. Signatures of selection on Z gametologs

We investigated whether the selection regime on Z gametologs was explained by the degree of degeneration of W gametologs and other putative predictors. As a response variable, we used dN/(dN + dS), calculated from the divergence with the Great tit, which reflects equal evolutionary times, but different proportions of time spent as sex-linked between strata (see Branch specific rate of sequence evolution). Genes with zero values of dN/(dN + dS) or missing data in any species were excluded. Genes in the PAR were included as an additional category to represent autosomal-like genes. Using *DHARMa*, we evaluated several potential models and transformations of response variables. The best fit was achieved with a Gaussian model, with the response variable log_10_-transformed to approximate a normal distribution. The model was built with *glmmTMB*, with the following predictors: W degeneration (categorical variable; 4 categories: functional, loss-of-function mutations, exon loss, and with PAR genes as a reference group), log_10_(gene length) (continuous variable), pHaplo (continuous variable) and species (categorical variable; 2 categories: Skylark and Raso lark). We excluded strata age (the number of generations since recombination cessation) due to co-linearity with the W degeneration (strata age: VIF = 5.83; W degeneration: VIF = 6.11). Similarly, stratum was tested as a predictor but was also highly co-linear with W degeneration (strata: VIF = 1.85E+6; W degeneration: VIF = 1.88E+6). After excluding these, no co-linearity remained (VIF ≤ 1.16). The model included all potential second-degree interactions. We performed model selection with *MuMIn dredge*, ranked by AIC. The top three models had AIC values < 2 and a cumulative weight of 0.655. Residuals of each model were evaluated with *DHARMa*. The first two models showed marginally significant KS tests (*P* = 0.0455; *P* = 0.0499), and all three models had significant adjusted quantile tests (*P* = 0.0181, *P* = 0.0164, *P* = 0.00467). The three models were averaged with *MuMIn model.avg*. The response variable was reported in its non-transformed format in all figures. Effect sizes were calculated as the weighted averages of standardised coefficients from each model. Functional relationships were visualised by plotting each variable while keeping all other predictors at the median of their distribution and averaging across categorical predictors. Further, we used the best model to estimate slopes and test contrasts between W degeneration categories along the full range of pHaplo scores using *emmeans*.

### 3j4. Signatures of selection on W gametologs

We investigated whether the selection regime on W gametologs could be explained by the strength of selection on Z gametologs and other predictors. As the response variable, we used dN/(dN + dS) since recombination cessation of W, and we used the same metric for Z gametologs as a predictor (See Branch specific rate of sequence evolution). Genes with zero values of dN/(dN + dS) were excluded. We included only genes where W was classified as functional or as carrying loss-of-function mutations. The response variable was log_10_-transformed to approximate a normal distribution. We built a Gaussian model with *glmmTMB*, including the following predictors: selection regime of Z (continuous variable), log_10_(gene length) (continuous variable), pHaplo (continuous variable), species (categorical variable; 2 categories: Skylark and Raso lark), strata age (continuous variable: time since recombination cessation in generations), and W functionality (categorical variable; 2 categories: functional and loss-of-function mutations). Co-linearity was assessed with *performance*, and no sign of co-linearity was detected (VIF ≤ 1.32). The model included all second-degree interactions. Model selection was performed with *MuMIn dredge*, ranked by AIC. The top 11 models had AIC values < 2 and a cumulative weight of 0.039. Residuals of each model were evaluated with *DHARMa*, and all models passed diagnostic checks. All 11 models were averaged with *MuMIn model.avg*. The response variable was reported in its non-transformed format in all figures. Effect sizes were calculated as weighted averages of standardised coefficients from each model. Functional relationships were visualised by plotting each variable, while keeping all other predictors at the median of their distribution and averaging across categorical predictors.

# 5. References

Alström P et al. 2023. Systematics of the avian family Alaudidae using multilocus and genomic data. Avian Res. 14:100095. https://doi.org/10.1016/j.avrs.2023.100095

Alström P, Ericson PGP, Olsson U, Sundberg P. 2006. Phylogeny and classification of the avian superfamily Sylvioidea. Mol Phylogenet Evol. 38(2):381–397. https://doi.org/10.1016/j.ympev.2005.05.015

Bartoń K. 2024. MuMIn: Multi-Model Inference. https://CRAN.R-project.org/package=MuMIn. https://doi.org/https://CRAN.R-project.org/package=MuMIn

Ben-Shachar MS, Lüdecke D, Makowski D. 2020. effectsize: Estimation of Effect Size Indices and Standardized Parameters. J Open Source Softw. 5(56):2815. https://doi.org/10.21105/joss.02815

Bird JP et al. 2020. Generation lengths of the world’s birds and their implications for extinction risk. Conserv Biol. 34(5):1252–1261. https://doi.org/10.1111/cobi.13486

Bolger AM, Lohse M, Usadel B. 2014. Trimmomatic: a flexible trimmer for Illumina sequence data. Bioinformatics. 30(15):2114–2120. https://doi.org/10.1093/bioinformatics/btu170

Brooks M E et al. 2017. glmmTMB Balances Speed and Flexibility Among Packages for Zero-inflated Generalized Linear Mixed Modeling. R J. 9(2):378. https://doi.org/10.32614/RJ-2017-066

Camacho C et al. 2009. BLAST+: architecture and applications. BMC Bioinformatics. 10(1):421. https://doi.org/10.1186/1471-2105-10-421

Castiglione S et al. 2018. A new method for testing evolutionary rate variation and shifts in phenotypic evolution Cooper N, editor. Methods Ecol Evol. 9(4):974–983. https://doi.org/10.1111/2041-210X.12954

Castresana J. 2000. Selection of Conserved Blocks from Multiple Alignments for Their Use in Phylogenetic Analysis. Mol Biol Evol. 17(4):540–552. https://doi.org/10.1093/oxfordjournals.molbev.a026334

Cingolani P et al. 2012. A program for annotating and predicting the effects of single nucleotide polymorphisms, SnpEff: SNPs in the genome of Drosophila melanogaster strain w ^1118^ ; iso-2; iso-3. Fly (Austin). 6(2):80–92. https://doi.org/10.4161/fly.19695

Collins RL et al. 2022. A cross-disorder dosage sensitivity map of the human genome. Cell. 185(16):3041-3055.e25. https://doi.org/10.1016/j.cell.2022.06.036

Cortez D et al. 2014. Origins and functional evolution of Y chromosomes across mammals. Nature. 508(7497):488–493. https://doi.org/10.1038/nature13151

Danecek P et al. 2011. The variant call format and VCFtools. Bioinformatics. 27(15):2156–2158. https://doi.org/10.1093/bioinformatics/btr330

Danecek P et al. 2021. Twelve years of SAMtools and BCFtools. GigaScience. 10(2):giab008. https://doi.org/10.1093/gigascience/giab008

Dierickx EG et al. 2020. Genetic diversity, demographic history and neo-sex chromosomes in the Critically Endangered Raso lark. Proc R Soc B Biol Sci. 287(1922):20192613. https://doi.org/10.1098/rspb.2019.2613

Durinck S, Spellman PT, Birney E, Huber W. 2009. Mapping identifiers for the integration of genomic datasets with the R/Bioconductor package biomaRt. Nat Protoc. 4(8):1184–1191. https://doi.org/10.1038/nprot.2009.97

Fregin S, Haase M, Olsson U, Alström P. 2012. New insights into family relationships within the avian superfamily Sylvioidea (Passeriformes) based on seven molecular markers. BMC Evol Biol. 12(1):157. https://doi.org/10.1186/1471-2148-12-157

Garrison E et al. 2022. A spectrum of free software tools for processing the VCF variant call format: vcflib, bio-vcf, cyvcf2, hts-nim and slivar Schneidman-Duhovny D, editor. PLOS Comput Biol. 18(5):e1009123. https://doi.org/10.1371/journal.pcbi.1009123

Garrison E, Marth G. 2012. Haplotype-based variant detection from short-read sequencing. arXiv. 1207.3907

Grabherr MG et al. 2010. Genome-wide synteny through highly sensitive sequence alignment: *Satsuma*. Bioinformatics. 26(9):1145–1151. https://doi.org/10.1093/bioinformatics/btq102

Hartig F. 2022. DHARMa: Residual Diagnostics for Hierarchical (Multi-Level / Mixed) Regression Models. http://florianhartig.github.io/DHARMa/. https://doi.org/http://florianhartig.github.io/DHARMa/

Hothorn T, Bretz F, Westfall P. 2008. Simultaneous Inference in General Parametric Models. Biom J. 50(3):346–363. https://doi.org/10.1002/bimj.200810425

Huang N, Lee I, Marcotte EM, Hurles ME. 2010. Characterising and Predicting Haploinsufficiency in the Human Genome Schierup MH, editor. PLoS Genet. 6(10):e1001154. https://doi.org/10.1371/journal.pgen.1001154

Jarvis ED et al. 2014. Whole-genome analyses resolve early branches in the tree of life of modern birds. Science. 346(6215):1320–1331. https://doi.org/10.1126/science.1253451

Kawakami T et al. 2014. A high‐density linkage map enables a second‐generation collared flycatcher genome assembly and reveals the patterns of avian recombination rate variation and chromosomal evolution. Mol Ecol. 23(16):4035–4058. https://doi.org/10.1111/mec.12810

Kinsella RJ et al. 2011. Ensembl BioMarts: a hub for data retrieval across taxonomic space. Database. 2011(0):bar030. https://doi.org/10.1093/database/bar030

Kosakovsky Pond SL et al. 2020. HyPhy 2.5—A Customizable Platform for Evolutionary Hypothesis Testing Using Phylogenies Crandall K, editor. Mol Biol Evol. 37(1):295–299. https://doi.org/10.1093/molbev/msz197

Lenth R. V. 2024. emmeans: Estimated Marginal Means, aka Least-Squares Means. https://rvlenth.github.io/emmeans/. https://doi.org/https://rvlenth.github.io/emmeans/

Li H et al. 2009. The Sequence Alignment/Map format and SAMtools. Bioinformatics. 25(16):2078–2079. https://doi.org/10.1093/bioinformatics/btp352

Li H. 2013. Aligning sequence reads, clone sequences and assembly contigs with BWA-MEM. arXiv. 1303.3997

Lüdecke D et al. 2021. performance: An R Package for Assessment, Comparison and Testing of Statistical Models. J Open Source Softw. 6(60):3139. https://doi.org/10.21105/joss.03139

MacArthur DG et al. 2012. A Systematic Survey of Loss-of-Function Variants in Human Protein-Coding Genes. Science. 335(6070):823–828. https://doi.org/10.1126/science.1215040

O’Leary SJ et al. 2018. These aren’t the loci you’e looking for: Principles of effective SNP filtering for molecular ecologists. Mol Ecol. 27(16):3193–3206. https://doi.org/10.1111/mec.14792

Oliveros CH et al. 2019. Earth history and the passerine superradiation. Proc Natl Acad Sci. 116(16):7916–7925. https://doi.org/10.1073/pnas.1813206116

Pala I et al. 2012. Evidence of a neo-sex chromosome in birds. Heredity. 108(3):264–272. https://doi.org/10.1038/hdy.2011.70

Paradis E, Schliep K. 2019. ape 5.0: an environment for modern phylogenetics and evolutionary analyses in R Schwartz R, editor. Bioinformatics. 35(3):526–528. https://doi.org/10.1093/bioinformatics/bty633

Purcell S et al. 2007. PLINK: A Tool Set for Whole-Genome Association and Population-Based Linkage Analyses. Am J Hum Genet. 81(3):559–575. https://doi.org/10.1086/519795

Ranwez V et al. 2018. MACSE v2: Toolkit for the Alignment of Coding Sequences Accounting for Frameshifts and Stop Codons Wilke C, editor. Mol Biol Evol. 35(10):2582–2584. https://doi.org/10.1093/molbev/msy159

Revell LJ. 2024. phytools 2.0: an updated R ecosystem for phylogenetic comparative methods (and other things). PeerJ. 12:e16505. https://doi.org/10.7717/peerj.16505

Rhie A et al. 2021. Towards complete and error-free genome assemblies of all vertebrate species. Nature. 592(7856):737–746. https://doi.org/10.1038/s41586-021-03451-0

Shen W, Le S, Li Y, Hu F. 2016. SeqKit: A Cross-Platform and Ultrafast Toolkit for FASTA/Q File Manipulation Zou Q, editor. PLOS ONE. 11(10):e0163962. https://doi.org/10.1371/journal.pone.0163962

Shumate A, Salzberg SL. 2021. Liftoff: accurate mapping of gene annotations Valencia A, editor. Bioinformatics. 37(12):1639–1643. https://doi.org/10.1093/bioinformatics/btaa1016

Sigeman H et al. 2019. Repeated sex chromosome evolution in vertebrates supported by expanded avian sex chromosomes. Proc R Soc B Biol Sci. 286(1916):20192051

Sigeman H et al. 2021. Avian Neo-Sex Chromosomes Reveal Dynamics of Recombination Suppression and W Degeneration Wilson M, editor. Mol Biol Evol. 38(12):5275–5291. https://doi.org/10.1093/molbev/msab277

Sigeman H, Sinclair B, Hansson B. 2022. Findzx: an automated pipeline for detecting and visualising sex chromosomes using whole-genome sequencing data. BMC Genomics. 23(1):328. https://doi.org/10.1186/s12864-022-08432-9

Sigeman H, Zhang H, Ali Abed S, Hansson B. 2022. A novel neo‐sex chromosome in *Sylvietta brachyura* (Macrosphenidae) adds to the extraordinary avian sex chromosome diversity among Sylvioidea songbirds. J Evol Biol. 35(12):1797–1805. https://doi.org/10.1111/jeb.14096

Smaers JB, Mongle CS, Kandler A. 2016. A multiple variance Brownian motion framework for estimating variable rates and inferring ancestral states. Biol J Linn Soc. 118(1):78–94. https://doi.org/10.1111/bij.12765

Smit A, Hubley R, Green P. 2013. RepeatMasker Open-4.0. https://doi.org/http://www.repeatmasker.org

Stiller J et al. 2024. Complexity of avian evolution revealed by family-level genomes. Nature. 629(8013):851–860. https://doi.org/10.1038/s41586-024-07323-1

Stoletzki N, Eyre-Walker A. 2011. Estimation of the Neutrality Index. Mol Biol Evol. 28(1):63–70. https://doi.org/10.1093/molbev/msq249

Talavera G, Castresana J. 2007. Improvement of Phylogenies after Removing Divergent and Ambiguously Aligned Blocks from Protein Sequence Alignments Kjer K, Page R, Sullivan J, editors. Syst Biol. 56(4):564–577. https://doi.org/10.1080/10635150701472164

Tan A, Abecasis GR, Kang HM. 2015. Unified representation of genetic variants. Bioinformatics. 31(13):2202–2204. https://doi.org/10.1093/bioinformatics/btv112

Warren WC et al. 2017. A New Chicken Genome Assembly Provides Insight into Avian Genome Structure. G3 GenesGenomesGenetics. 7(1):109–117. https://doi.org/10.1534/g3.116.035923

Wright AE, Moghadam HK, Mank JE. 2012. Trade-off Between Selection for Dosage Compensation and Masculinization on the Avian Z Chromosome. Genetics. 192(4):1433–1445. https://doi.org/10.1534/genetics.112.145102

Xu L et al. 2019. Dynamic evolutionary history and gene content of sex chromosomes across diverse songbirds. Nat Ecol Evol. 3(5):834–844. https://doi.org/10.1038/s41559-019-0850-1

Yazdi HP et al. 2023. The evolutionary maintenance of ancient recombining sex chromosomes in the ostrich Bosco G, editor. PLOS Genet. 19(6):e1010801. https://doi.org/10.1371/journal.pgen.1010801

Yu G, Wang L-G, Han Y, He Q-Y. 2012. clusterProfiler: an R Package for Comparing Biological Themes Among Gene Clusters. OMICS J Integr Biol. 16(5):284–287. https://doi.org/10.1089/omi.2011.0118

Zamani N et al. 2014. A universal genomic coordinate translator for comparative genomics. BMC Bioinformatics. 15(1):227. https://doi.org/10.1186/1471-2105-15-227

Zhang C et al. 2019. PopLDdecay: a fast and effective tool for linkage disequilibrium decay analysis based on variant call format files Schwartz R, editor. Bioinformatics. 35(10):1786–1788. https://doi.org/10.1093/bioinformatics/bty875

Zhou Q et al. 2014. Complex evolutionary trajectories of sex chromosomes across bird taxa. Science. 346(6215):1246338. https://doi.org/10.1126/science.1246338
