# Supplementary material for: Selective Regimes and Evolutionary Dynamics of Z and W Gametologs Across an Expanded Avian Neo-Sex Chromosome
Source: Genome Biol Evol. 2026 Aug 10;18(8):evag204. doi: 10.1093/gbe/evag204 (PMC13524101; doi:10.1093/gbe/evag204)
Supplement: Supplementary file 1 [file evag204_supplementary_data.zip › Supplement_260803.docx]

Supplementary files: Figure S1-S15

**Manuscript Title:**

Selective regimes and evolutionary dynamics of Z and W gametologs across an expanded avian neo-sex chromosome

**Author information:**

Simon J Ellerstrand^1^, Bengt Hansson^1^

^1^Department of Biology, Evolutionary Ecology and Infection Biology, Science for Life Laboratory, Lund University, Ecology Building, 223 62 Lund, Sweden

ORCID:

SJE 0000-0003-2674-6997
BH 0000-0001-6694-8169

Contents

[Figure S1-S3: PhaseWY pipeline performance	2](#_Toc236655860)

[Figure S4: Species variation in PAR boundary on stratum 3-c	5](#_Toc236655861)

[Figure S5-S6: Generation time and age of evolutionary strata	6](#_Toc236655862)

[Figure S7: Functional degeneration across genomic regions and species	8](#_Toc236655863)

[Figure S8-9: Predictors of W degeneration	9](#_Toc236655864)

[Figure S10-13: Signatures of selection on Z gametologs	11](#_Toc236655865)

[Figure S14: Signatures of selection on W gametologs	15](#_Toc236655866)

[Figure S15: Branch-specific rates of sequence evolution	16](#_Toc236655867)

## Figure S1-S3: PhaseWY pipeline performance

**Figure S1.** Principal Component Analysis (PCA) results showing PC1 and PC2 for male (blue) and female (red) Skylarks and Raso larks. (**A, B**) PCA based on all variants before applying PhaseWY. (**C, D**) PCA based on coding autosomal sequences, and (**E, F**) coding Z sequences, following genome classification and data subsetting with PhaseWY.

**Figure S2.** Folded Site Frequency Spectrum (SFS) for Skylark and Raso lark (sexes combined). (**A, B**) SFS based on all variants prior to PhaseWY processing. (**C, D**) SFS of coding autosomal sequences, and (**E, F**) of coding Z sequences, following genome classification and data subsetting with PhaseWY. Dashed red line indicates the frequency where W-linked variants may inflate the distribution if present in the dataset, as is clearly observed in the Raso lark, and dataset including all variants prior to PhaseWY processing (B).

**Figure S3.** The inbreeding coefficient (F_IS_) of male (blue) and female (red) Skylarks and Raso larks. (**A, B**) F_IS_ based on all variants prior to PhaseWY processing. (**C, D**) F_IS_ based on coding autosomal sequences following genome classification and data subsetting with PhaseWY. Results for the Z and W chromosomes are not shown, as F_IS_ is only applicable to diploid data.

## Figure S4: Species variation in PAR boundary on stratum 3-c

**Figure S4. Species variation in the PAR boundary of stratum 3-c.** PhaseWY classification of regions in coordinates on Skylark scaffold CADDXX010000137.1 in **(A)** the Skylark, and **(B)** the Raso lark. A putative rearrangement is marked with a black line between panels. **(C)** Location of genes with names based on the Zebra finch annotation. **(D)** Absolute divergence (D_XY_) between Skylark and Raso lark for W sequences, and for Z and PAR sequences. Divergence is expected to be higher in sex-linked regions due to drift, especially in comparisons involving the W and the bottlenecked Raso lark. Note that comparisons involving the W chromosome only extends as far as the span of the W-specific region. **(E)** Linkage disequilibrium (LD) in female Skylarks and Raso larks, respectively. This analysis is based on non-subset data, implying that both Z and W sequences are present. Therefore, elevated LD is expected in sex-linked regions, while lower values are expected in the PAR.

## Figure S5-S6: Generation time and age of evolutionary strata

**Figure S5.** Estimated ancestral generation lengths in birds, modelled as a branch trait and visualised on the dated phylogeny from Stiller et al., (2024). Branch colours represent estimated generation times, with warm colours (red) indicating shorter, and cold colours (blue) longer, generation lengths.

**Figure S6.** Relationship between phylogenetic node age measured in generations (million generations) and years (million years), respectively. Blue circles represent dated nodes traced from the Skylark to the root of the dated phylogeny from Stiller et al., (2024). The black line shows the fitted polynomial function based on these nodes (R^2^=0.9993). Red triangles indicate predicted ages of strata, derived from the fitted model.

## Figure S7: Functional degeneration across genomic regions and species

**Figure S7.** Proportion of functional and degenerated genes across genomic regions. Genes located on autosomes, PAR and Z are classified into two categories based on predicted mutational impact on protein using SnpEff: genes without loss-of-function mutations (“functional”; light blue) and genes with loss-of-function mutations (“loss-of-function mutations”; yellow). W gametologs are further divided into two additional categories based on female-to-male (ZW vs. ZZ) read depth differences, which indicate W sequence loss: partial loss of exons (“partial exon loss”; orange) and complete loss of exons (“Full exon loss”; red).

## Figure S8-9: Predictors of W degeneration

**Figure S8.** Effect sizes (log10 of odds ratios) for all predictors in the model describing the probability of W-gene non-functionality. Estimates are derived from a binomial generalized linear model (GLM) of W gametologs classified as either functional (genes without loss-of-function mutations) or non-functional (genes with loss-of-function mutations, or with exon loss).

**Figure S9.** Predicted probability of non-functionality in larks (Alauda) as a function of stratum age (in million generations, MG), with other predictors held at their median values. Points represent the observed proportion of non-functional genes on each stratum (and PAR 3 and 5 combined at age zero). In addition, empirical values from Krasovec et al. (2018) are added, with model fit using a quasibinomial GLM with a logit link in ggplot2.

## Figure S10-13: Signatures of selection on Z gametologs

**Figure S10.** Effect sizes (β) of predictors of Z selection strength. PAR genes serve as the reference category for the W functional–degeneration predictor. Estimates are derived from a linear model (LM) of strength of selection since divergence from the Great tit.

**Figure S11.** Distribution and count of DoS (Direction of Selection) in genes in PAR and on Z, depending on W functional status. Coloured points and dashed lines represent group medians (PAR=0.0237, W functional=0.0183, W loss-of-function mutation=0.0537, W exon loss=0.0365).

**Figure S12.** Distribution and count of pHaplo (haploinsufficiency) in genes in PAR and on Z, depending on W functional status. Coloured points represent group medians.

**Figure S13.** Signatures of selection in the outgroup autosomal lineage (representing the divergence of the Great tit relative to Zebra finch and Collared flycatcher), in relation with haploinsufficiency in genes in PAR and on Z, depending on W functional status (±95% CI). Purifying selection strengthens (lower dN/(dN + dS)) with increasing haploinsufficiency (high pHaplo).

## Figure S14: Signatures of selection on W gametologs

**Figure S14.** Effect sizes (β) of predictors of W selection strength. W functional serve as the reference category for the W functionality predictor. Estimates are derived from a linear model (LM) of strength of selection since onset of recombination cessation for respective gene.

## Figure S15: Branch-specific rates of sequence evolution

**Figure S15.** Maximum likelihood estimates of branch-specific dN/dS (Omega) and branch lengths from the MG94 model. Estimates of rates and branch lengths were calculated as weighted averages across genes, using quality-trimmed gene length as weights. All rates are presented as the log_10_-transformed dN/dS values. Branch-specific rates and lengths are shown for genes located on all neo-sex chromosome strata, as well as for each independent stratum. Abbreviations: SW=Skylark W, RW=Raso lark W, SZ=Skylark Z, RZ=Raso lark Z, GTA=Great tit autosomal, ZFA=Zebra finch autosomal, CFA=Collared flycatcher autosomal. Tips represent Z and W sequences of individual females.
